# Enhanced Anticancer Effect of a Combination of S-adenosylmethionine (SAM) and Immune Checkpoint Inhibitor (ICPi) in a Syngeneic Mouse Model of Advanced Melanoma

**DOI:** 10.3389/fonc.2020.01361

**Published:** 2020-09-02

**Authors:** Ali Mehdi, Mikhael Attias, Niaz Mahmood, Ani Arakelian, Catalin Mihalcioiu, Ciriaco A. Piccirillo, Moshe Szyf, Shafaat Ahmed Rabbani

**Affiliations:** ^1^Department of Medicine, McGill University, Montreal, QC, Canada; ^2^Human Genetics, McGill University, Montreal, QC, Canada; ^3^Program in Metabolic Disorders and Complications (MeDiC), Research Institute of the McGill University Health Centre, Montreal, QC, Canada; ^4^Microbiology & Immunology, McGill University, Montreal, QC, Canada; ^5^Experimental Medicine, McGill University, Montreal, QC, Canada; ^6^Department of Oncology, McGill University, Montreal, QC, Canada; ^7^Program in Infectious Diseases and Immunology in Global Health, Centre for Translational Biology, Research Institute of the McGill University Health Centre, Montreal, QC, Canada; ^8^Centre of Excellence in Translational Immunology (CETI), Montreal, QC, Canada; ^9^Department of Pharmacology, McGill University, Montreal, QC, Canada

**Keywords:** DNA methylation, melanoma, S-adenosylmethionine, anti-PD-1, immunity, immune checkpoint inhibitors

## Abstract

Immune checkpoint inhibitors (ICPi) targeting the PD-1/PD-L1 pathway have shown marked success in patients with advanced melanoma. However, 60–70% of patients fail to respond, warranting a therapeutic intervention that could increase response rates. We and others have shown that S-adenosylmethionine (SAM), a universal methyl donor, has significant anticancer effects in numerous cancers previously; however, its effect on melanoma progression has not been evaluated. Interestingly, SAM was reported to be essential for T cell activation and proliferation and, thus, could potentially cooperate with ICPi and block melanoma progression. In this study, we examined the antitumor effects of SAM and ICPi alone and in combination in a well-established melanoma mouse model wherein syngeneic C57BL/6 mouse were subcutaneously (orthotopic) injected with B16-F1 cells. Treatment of mice with either SAM or anti-PD-1 antibody alone resulted in significant reduction in tumor volumes and weights; effects that were highest in mice treated with a combination of SAM+anti-PD-1. RNA-sequencing analysis of the primary tumors showed numerous differentially expressed genes (DEGs) following treatment with SAM+anti-PD-1, which was shown to downregulate cancer, MAPK, and tyrosine kinase pathways. Indeed, SAM+anti-PD-1 reversed the aberrant expression of some known melanoma genes. Tumor immunophenotyping revealed the SAM+anti-PD-1 combination was significantly more effective than either SAM or anti-PD-1 as the CD8^+^ T cells had higher activation, proliferation, and cytokine production compared to all other groups. This study shows that the combination of currently approved agents SAM and ICPi can effectively block melanoma via alteration of key genes/pathways implicated in cancer and immune response pathways, providing the rationale for the initiation of clinical trials with SAM and ICPi.

## Introduction

Melanoma has one of the top 10 incidence rates among tumor types, causing high rates of mortality and warranting an urgent need for the development of new innovative therapeutic strategies, particularly for patients with advanced melanoma for whom treatment options are very limited (1).

Epigenetic deregulation of gene transcription via DNA methylation, histone modification, and non-coding RNA is a common heritable mechanism in many cancers, including melanoma, which can alter the expression of key genes implicated in tumor progression (2). The first report of “substantial hypomethylation” of CpG dinucleotides present in human cancer cells was published in 1983 (3). Since then, numerous studies have shown that, typically in cancer, genome-wide global DNA hypomethylation occurs in cancer, which contributes to genomic instability and activation of silenced oncogenes (4). S-adenosylmethionine (SAM) is synthesized endogenously and acts as a methyl group donor in DNA methylation reactions and has also been approved as a nutraceutical agent (5, 6). SAM treatment has significant anticancer effects on breast, osteosarcoma, prostate, hepatocellular, gastric, colon, and other cancer models (6–10). SAM effectively reduces cancer proliferation and metastasis by inhibiting angiogenesis, reducing inflammation, and downregulating several genes involved in promoting cell proliferation, invasion, and metastasis (5–12). For instance, we reported that the antimetastatic activity of SAM in breast and prostate cancer is likely due to downregulation of pro-metastatic genes, such as urokinase plasminogen activator (*uPA*) and Matrix metallopeptidase 2 (*MMP2*) (6, 9). It is unknown whether SAM has similar effects on melanoma. SAM has also been reported to be required for activation and proliferation of T cells (13–16). In activated T cells, both SAM levels and the rate of its utilization increase although inhibition of SAM synthesis results in reduced T cell proliferation (13–16). However, the role of SAM in cancer immunity has not been yet examined.

An important step involved in melanoma progression is immune evasion. A major pathway through which tumors induce immunosuppression involves binding of programmed death ligand 1 (PD-L1), expressed on the surface of melanoma cells, on to its receptor programmed cell death 1 (PD-1), a coinhibitory surface checkpoint receptor on T cells (17, 18). PD-1 signaling results in inhibition of T cell proliferation, cytokine production, production of anti-apoptotic molecules, and a metabolic shift that amounts to a state of exhaustion (17–20). Immune checkpoint inhibitors (ICPi), such as anti-PD-1 and anti-PD-L1 monoclonal antibodies, reverse this immunologically tolerant state and induce tumor regression in responding patients (1, 18, 20). Apart from metastatic melanoma, the FDA has approved ICPi as a frontline treatment of multiple cancers, including non-small cell lung cancer (NSCLC), renal cell carcinoma (RCCs), and bladder or urothelial cancer (1, 18, 20). However, there is significant variability in response to ICPi therapy, and 60–70% of patients fail to respond to single-agent ICPi therapy (1, 18, 20–22). Thus, there is a need to develop innovative approaches to enhance the response to ICPi monotherapy.

Epigenetic drugs are a class of agents that could potentially enhance ICPi anticancer activity by altering the epigenetic programming of genes that mediate the checkpoint response in the immune system and the cellular responses in cancer cells. Both clinical studies and animal models have shown that some epigenetic drugs prime the immune system and upregulate expression of immune-response signaling pathways in cancer cells, thereby improving immune recognition and immunogenicity (10, 23, 24). SAM being a methylating agent could lead to alterations in the expression of immune related genes, which could increase immunogenicity of the tumors. Also, SAM, known for its anticancer effects in various cancers and an immune regulator essential for T cell activation and proliferation, could, thus, provide a superior anticancer effect when combined with ICPi. In this report, we tested first whether SAM would have anticancer effects in melanoma, second whether a combination of SAM and ICPi would have an enhanced antitumor effect, and third we delineated the molecular pathways affected by the combination in comparison to monotherapy with either ICPi or SAM.

## Materials and Methods

### Cell Lines

The B16-F1 mouse melanoma cell line (CRL-6323™) was obtained from the American Type Culture Collection (ATCC; Manassas, Virginia). Cells were cultured in Dulbecco's modified Eagle's medium (DMEM) supplemented with 10% fetal bovine serum (FBS), 1% penicillin-streptomycin sulfate, and 2 mM L-glutamine. The cells were maintained in incubators at 37°C and 5% CO_2_ and were found to be mycoplasma free.

### Proliferation, Colony Formation and Invasion Assays

For *in vitro* efficacy, we used 200 μM of SAM (catalog #B9003S, New England Biolabs, Canada), which was found to be the optimum dose in our previous studies and following the evaluation of different doses of SAM in B16-F1 cells and 50 μg/mL of anti-PD-L1 (clone 10F.9G2, catalog #BE0101, BioXcell, USA) (6–9, 25, 26). B16-F1 cells (2 × 10^4^ cells) were seeded in 6-well plates. The experiment had five treatment groups; No rPD-1 (control without rPD-1), rPD-1 control (Control with rPD-1), SAM, anti-PD-L1, and SAM+anti-PD-L1, and cells in these wells were treated accordingly. B16-F1 cells were stimulated with 0.2 μM rPD-1 (catalog #1021-PD-100, R&D systems, USA) on day 3 to stimulate the PD-1/PD-L1 pathway before adding 50 μg anti-PD-L1 on day 4, 200 μM of SAM was added on days 2–4, and cells were harvested on day 5. Each experiment was carried out in duplicate.

For the proliferation assay, cells on day 5 were trypsinized and counted using the Beckman Coulter counter (Model ZF; Coulter Electronics, Hertfordshire, UK) according to the manufacturer's instructions. Proliferation assay results are the mean of four independent experiments performed in duplicate. Results are presented as the percentage of proportion to the rPD-1 Control ± SEM.

For the colony formation assay, after following the proliferation assay protocol, 5,000 treated cells in DMEM (13% FBS) were mixed with agar in a 3:1 ratio and poured into a well of 6-well plates until solidified, followed by adding 2 mL of DMEM on top. Colonies were monitored and counted after 2 weeks. Data is presented as mean number of colonies ± SEM.

Following the proliferation assay protocol, the invasion assay was performed as previously described by us using a two-compartment Boyden chamber invasion assay (Costar Transwell, Corning Corporation, Sigma-Aldrich, Oakville, ON, Canada) (6). The precise steps for the invasion assay are detailed in the previous paper (6) except that the B16-F1 cells were incubated for 24 h instead of 18 h. Data is presented as mean number of cells invaded per field ± SEM.

### Animal Studies

All *in vivo* studies were performed in accordance with McGill University Facility Animal Care Committee guidelines. Six- to eight-week-old female C57BL/6 or Black B6 mice were purchased from Charles River Lab (Quebec, Canada) and housed at the Animal Resource Division (ARD) of the Research Institute of the McGill University Health Center (RI-MUHC). To determine the effect of SAM (Life Science Laboratories, Lakewood, NJ, USA), anti-PD-1 (clone RMP1-14, BioXcell, USA), and SAM+anti-PD-1 combination on tumor growth, mice were injected orthotopically with 5 × 10^5^ B16-F1 cells via the subcutaneous (s.c) route into the left flank to induce tumor formation. These mice were randomized into the four groups and then treated with either isotype-matched control IgG (control), SAM, anti-PD-1 and SAM+anti-PD-1 combination (*n* = 8 per group). Treatment was started at day 3 wherein 80 mg/kg of SAM diluted in PBS was given daily via oral gavage using feeding needles, and 10 mg/kg anti-PD-1 was given via intraperitoneal (i.p.) injection twice a week with a total of four doses of anti-PD-1. The dose of SAM 80 mg/kg was established in our previous study (6), and the dose of anti-PD-1, 10 mg/kg, was established previously in preclinical and clinical trails (20, 27–32). Tumor volumes were measured by palpation at days 12 and 14 using a caliper. On day 16, mice were sacrificed, and tumor weight (T.W.) and tumor volumes (T.V.) were measured and calculated using the formula T.V. = (length × width^2^)/2. Percentage (%) of tumor reduction at day 16 was calculated as [(mean T.V. or T.W. of (control-treatment group))/mean T.V. or T.W. of control] ^*^ 100. The animals were weighed at the start of the study and at the time of tumor volume measurement. Regular examinations were carried out for any body weight loss or potential adverse effect as we have previously reported in the B16 melanoma model (33). Due to low viability of tumor-infiltrating cells at the humane end point, pilot studies were performed to determine the optimal experimental end point for detection of immune cell populations in the tumor microenvironment of B16-tumor inoculated B6 mice. For immunophenotyping experiments, we selected day 14 as our experimental end point and used the SAM treatment arms and dosage; however, mice receiving anti-PD-1 were injected with a total of three injections post-tumor inoculation.

### RNA Extraction and Reverse Transcriptase Quantitative Real-Time PCR (RT-qPCR)

Total cellular and tumoral RNA was extracted using the RNeasy kit (Qiagen; Hilden, Germany, Cat# 71404) according to the manufacturer's instructions. The RT-qPCR assay was performed following our previously described protocol (6). The primers are listed in Supplementary Table 1. Change in gene expression among the various groups was analyzed by using the 2-ΔΔCT method.

### RNA Sequencing (RNA-Seq)

Total RNA from the cells and tumors was extracted as described above. The extracted RNA was sent to the Genome Quebec and Innovation Centre (McGill University) for carrying out paired-end RNA sequencing using the Illumina HiSeq 4,000 platform (with a depth of 50 million reads) following standard protocols. The obtained data was analyzed using DeSeq2 script in R according to the writer's recommendations (34).

### Immunophenotyping

Mice (*n* = 8/group from two independent experiments) were sacrificed at day 14, and primary tumors, spleens, and lymph nodes (draining and contralateral) were harvested and placed in RPMI 1,640 1× (Wisent, Saint-Jean-Baptiste QC, Canada; Cat# 319-015-CL). Spleens and lymph nodes were dissociated mechanically into single cell suspensions. Whole tumors were shredded thinly, before digestion with collagenase IV (Gibco) and DNAse I (Sigma-Aldrich) for 1 h at 37°C. Cells were then passed through a 70-μm cell strainer to obtain single-cell suspensions. After lymphocyte isolation, the cells were then washed in PBS and stained first with antiCD16/CD32 (clone 2.4G2, BD) and then extracellular marked, fixed, and permeabilized for intracellular staining, followed by flow cytometry analysis. For assessment of cytokine production, single-cell suspensions were stimulated with Phorbol 12-myristate 13-acetate (PMA), Ionomycin, and incubated in the presence of GolgiStop (BD Biosciences) for 3 h at 37°C before staining for flow cytometry analysis. Samples were acquired using the BD Fortessa LSR-X20 and analyzed using FlowJo v10 (TreeStar) (35). The fluorescence-conjugated antibodies used for staining are listed in Supplementary Table 2.

### Statistical Analysis

Results were analyzed and presented as ± SEM or SD, statistical difference between different groups determined by two-tailed Student's *t*-test and one-way ANOVA, where values of ^*^P < 0.05, ^**^P < 0.01, ^***^P < 0.001, ^****^P < 0.0001 were considered statistically significant. For gene set enrichment analysis, Consensus PathDB was used (36).

## Results

### Effect of SAM and Anti PD-L1 Antibody on B16 Melanoma Cell Proliferation, Colony Formation, and Invasion *in vitro*

**Figure 1:**
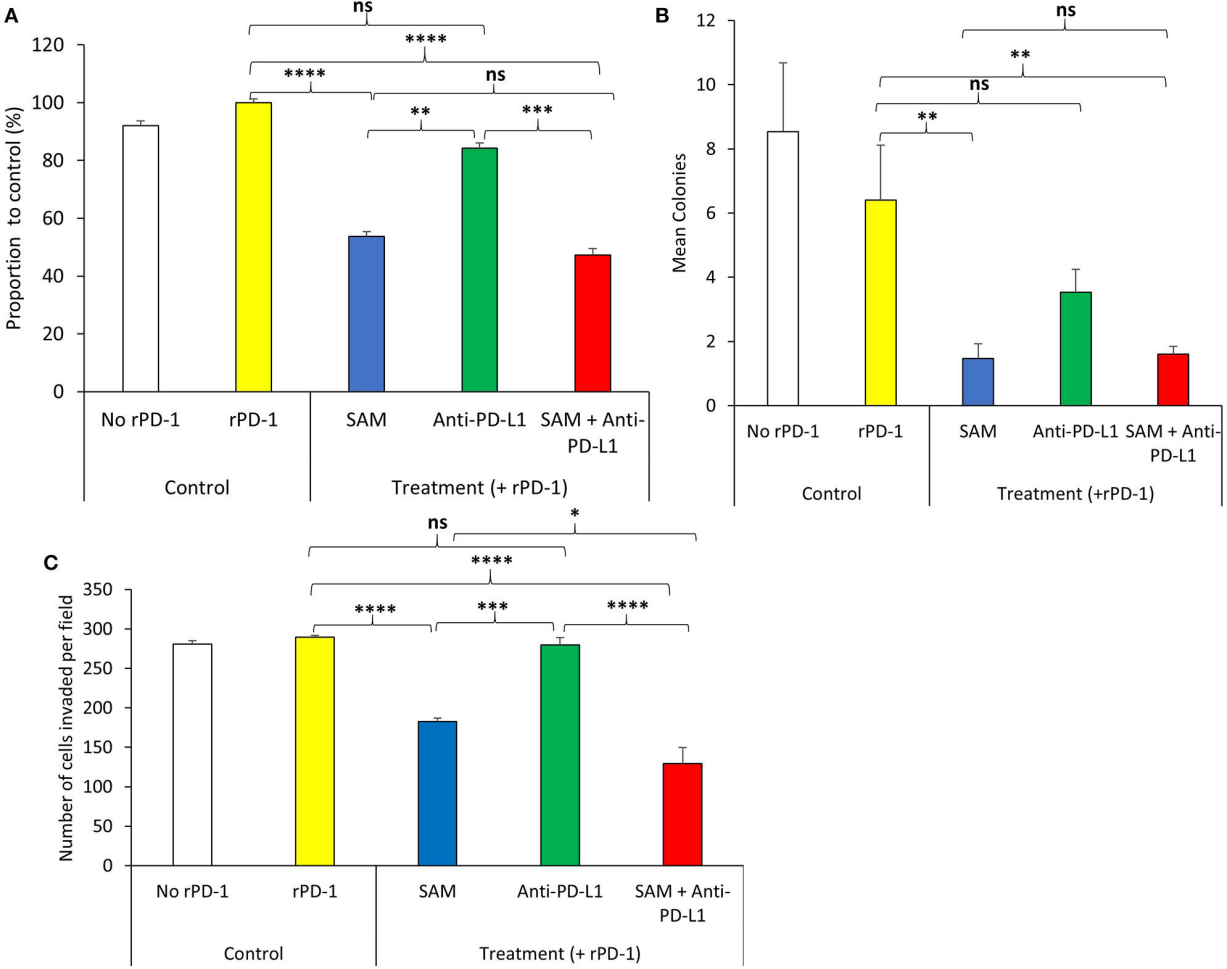
Effect of SAM and anti-PD-L1 antibody on B16-F1 melanoma cell proliferation, colony formation, and invasion *in vitro*. B16-F1 cells (2 × 10^4^ cells) were seeded in 6-well plates and were stimulated with rPD-1. The experiment had five treatment groups: No rPD-1 added (control no rPD-1), 0.2 μM rPD-1 control (Control with rPD-1), treated with 0.2 μM rPD-1 followed by treatment with 200 μM SAM (SAM), 50 μg/mL of anti-PD-L1, or combination of SAM+anti-PD-L1, and cells were subjected to proliferation, colony formation, and invasion assay as described in Materials and Methods. **(A)** Proliferation is presented as the percentage of rPD-1 Control ± SEM. **(B)** Colony formation is presented as mean ± SEM. **(C)** Invasion assay is presented as mean number of cells invaded per field ± SEM. Results are mean of at least two independent experiments. Statistical significance was determined by ANOVA in GraphPad prism and are represented by asterisks (ns, not significant, *P < 0.05; **P < 0.01, ***P < 0.001, and ****P < 0.0001).

SAM has been reported to have significant anticancer effects both *in vitro* and *in vivo* in several cancers; however, the effect of SAM has not been tested on melanoma yet (6–10). We first investigated the effect of increasing doses of SAM on B16-F1 cell proliferation, where 200 μM was most effective in reducing cell proliferation (Supplementary Figure 1). The maximum anticancer effects of SAM were seen following treatment with 200 μM, and no additional increment was seen with a higher dose of SAM. Although it is established that the major anticancer effects of blockage of the PD-1/PD-L1 pathway are related to enhancing immunity against cancer, there are various reports that PD-L1 triggers intrinsic signaling independent of the immune checkpoint, which promotes tumorigenesis (37, 38). Hence, we determined the effect of SAM and anti-PD-L1 in an *in vitro* cell proliferation assay. Because PD-1 is not present in an *in vitro* system, we used recombinant PD-1 (r-PD-1) to stimulate the intracellular PD-1/PD-L1 pathway. As in other cancer cell line models, SAM treatment resulted in a significant decrease in B16-F1 melanoma cell proliferation. Although anti-PD-L1 showed a slight decrease in cell proliferation, it was not statistically significant; however, combination of SAM+anti-PD-L1 showed significantly higher reduction in cell proliferation compared to control (Figure 1A). A similar pattern was observed for B16-F1 cells in a colony formation assay, where the lowest number of colonies were seen following treatment with SAM+anti-PD-L1 in combination setting (Figure 1B). The number of invasive cells were significantly lower in the combination of SAM+anti-PD-L1 group compared to all the other groups (Figure 1C). Collectively, these results show that SAM but not anti-PD-L1 decreased cell proliferation, anchorage-independent growth, and invasive ability of B16-F1 melanoma cells *in vitro*. These results provide evidence that SAM is effective in blocking melanoma cell proliferation, colony formation, and invasion *in vitro*, results that are similar to our and others' previous studies in several cancer cell lines (6–12).

### Effect of SAM and Anti-PD-1 Antibody Alone and Their Combination on Tumor Growth in a Syngeneic B16-F1 Mouse Melanoma Model

**Figure 2:**
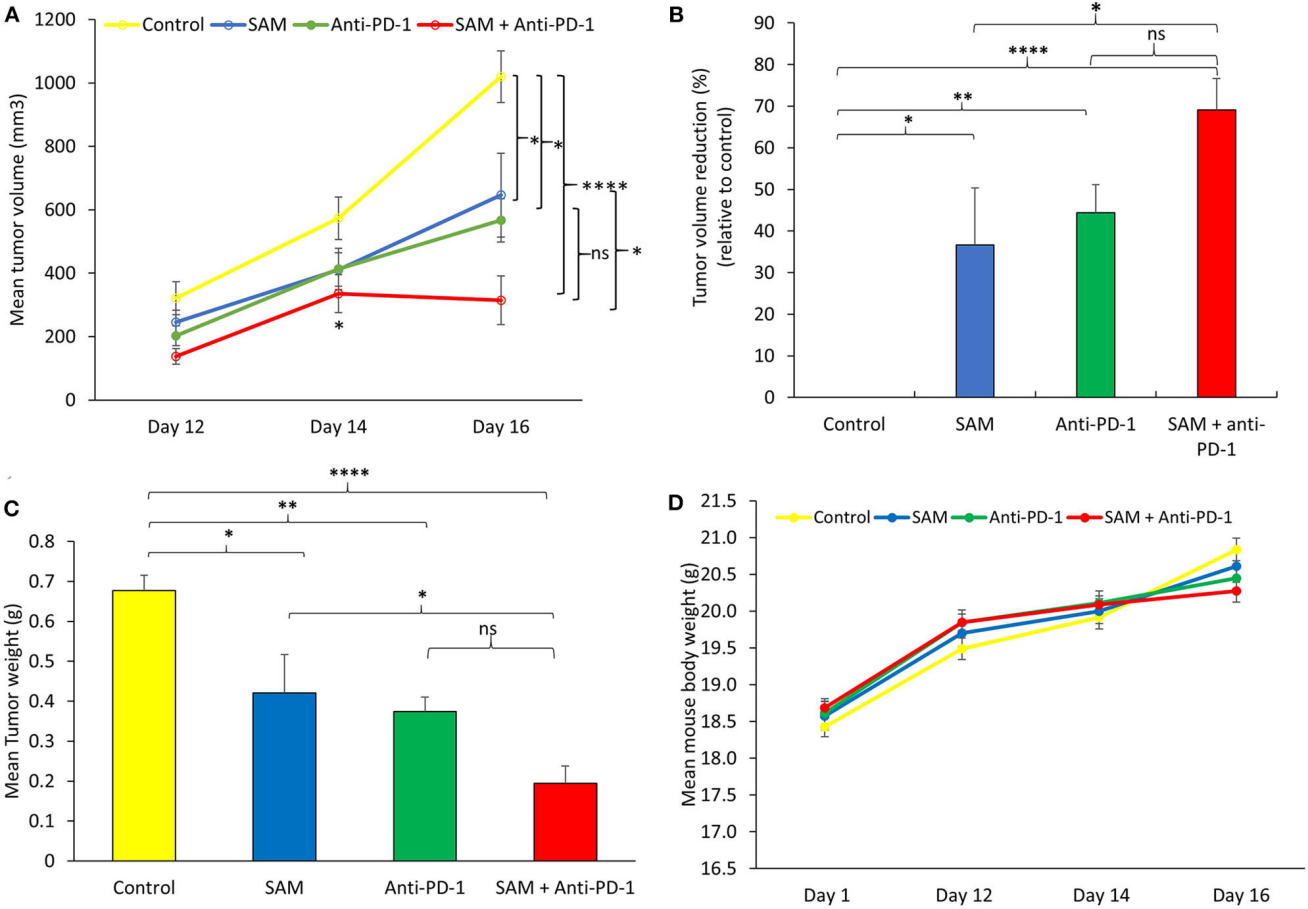
Antitumor effect of SAM and anti-PD-1 antibody in a syngeneic mouse B16-F1 melanoma model *in vivo*. 5 × 10^5^ B16-F1 mouse melanoma cells were injected via the subcutaneous route into the right flank of C57BL/6 mouse (*n* = 8/group). From day 3 post tumor cells, inoculation mice were treated with control IgG alone (control), 80 mg/kg SAM, 10 mg/kg anti-PD-1 antibody, and SAM+anti-PD-1 antibody as described in Materials and Methods. **(A)** Tumor volume was measured at days 12, 14, and 16. All control and experimental mice were sacrificed on day 16. **(B)** Percentage (%) of tumor volume reduction in each group relative to control at day 16. **(C)** Tumor weight was measured after sacrifice on day 16. **(D)** Mean mouse body weights measured at different time intervals (days 1, 12, 14, and 16) for each group. **(A–D)** Results are representative of mean ± SEM of at least 8 mice per group. Statistical significance was obtained by ANOVA in GraphPad prism and are represented by asterisks *(*ns, not significant, *P < 0.05, **P < 0.01, and ****P < 0.0001).

Next, we examined the effect of SAM and anti-PD-1 and the combination of both agents in B16-F1 melanoma-bearing mice. Using this syngeneic cell line approach, immunocompetent mice develop a failing adaptive immune response that does not stop tumor growth. This model has been widely used for preclinical assessment of antimelanoma immunotherapies (39, 40). B16-F1 melanoma cells were injected via the subcutaneous (s.c.) route into female C57BL/6 mice followed by treatment with either control IgG, SAM, anti-PD-1 antibody, or SAM+anti-PD-1 antibody. Tumor volumes were measured at timed intervals (days 12 and 14), and at the end of this study on day 16, all control and experimental mice were sacrificed. In this model of aggressive advanced melanoma, all three treatment arms had statistically significant reduced tumor burdens compared to the controls (SAM, 646 mm^3^, *p* < 0.05; anti-PD-1, 567 mm^3^*, p* < 0.05; and control 1,020 mm^3^), whereas the combination group of SAM+anti-PD-1 had significantly lower mean tumor volume (315 mm^3^) relative to control (*p* < 0.0001) and SAM (*p* < 0.05) at the end point (Figure 2A). Moreover, in the SAM+anti-PD-1 group, there was no measurable increase in mean tumor volume between days 14 and 16 (Figure 2A). Additionally, the SAM+anti-PD-1 treated group had the highest percentage of tumor volume reduction (69%, *p* < 0.0001) relative to control as compared to SAM and anti-PD-1 alone (37 and 44%, respectively) at the end point (Figure 2B). Similarly, all three treatment arms had significantly lower mean tumor weight compared to control (SAM, 0.42 g, *p* < 0.05; anti-PD-1, 0.37 g, *p* < 0.01; and control, 0.68 g), and the SAM+anti-PD-1 group had significantly lower mean tumor weight (0.20 g) compared to control (*p* < 0.0001) and SAM (*p* < 0.05) (Figure 2C). The percentage tumor weight reduction was also significantly lower for the SAM+anti-PD-1 group (71%) relative to control (*p* < 0.0001) and SAM (39%, *p* < 0.05) (Supplementary Figure 2). Regular examinations of control and experimental groups of animals showed no significant (*p* > 0.05) body weight loss following all treatments (Figure 2D). These data support the benefit of a combination of SAM+anti-PD-1 for inhibiting melanoma growth and progression as compared to SAM and anti-PD-1 as a monotherapy.

### Effect of Combined SAM+Anti PD-1 Therapy on the Transcriptional Landscape of B16-F1 Tumors

**Figure 3:**
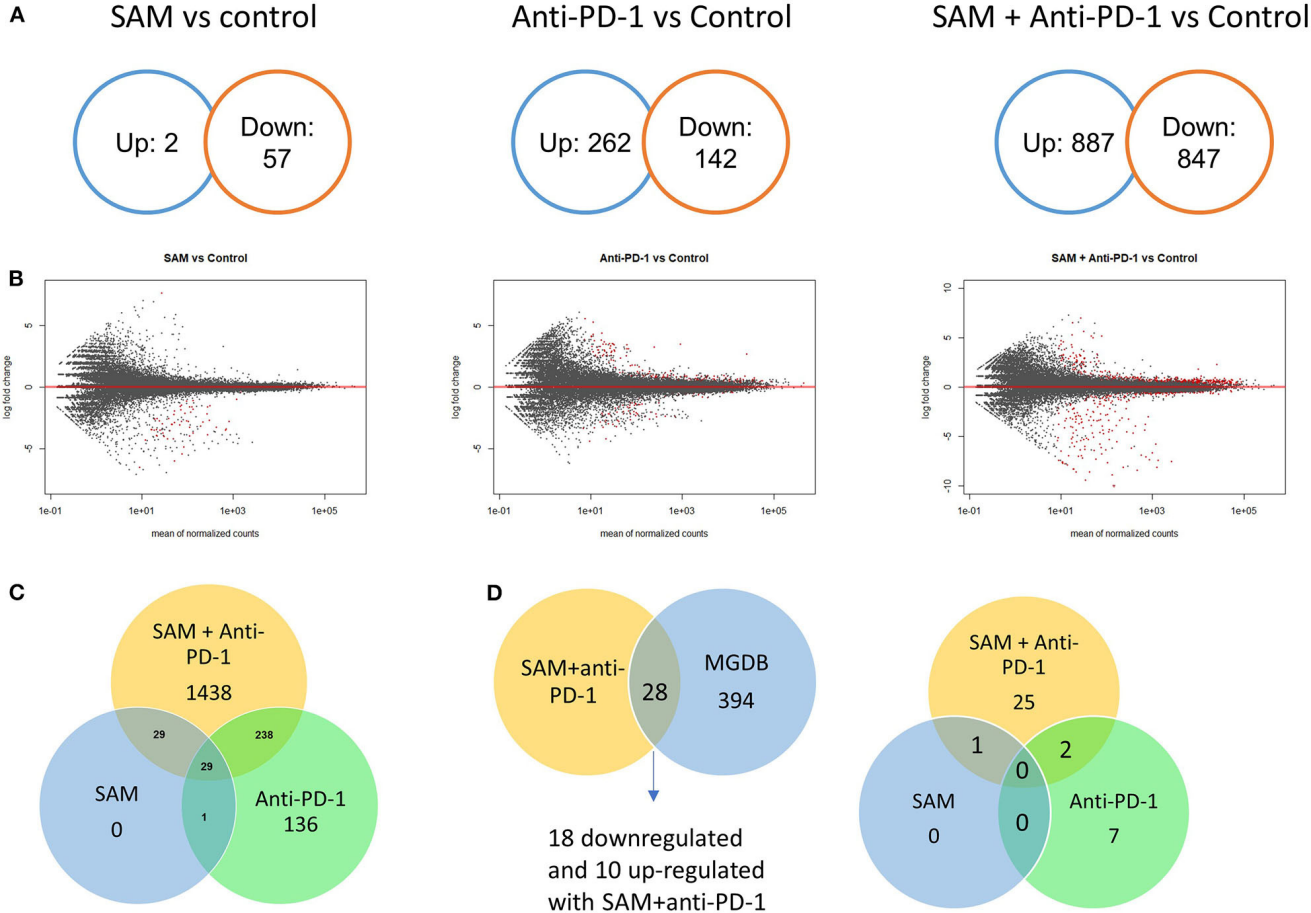
Transcriptome analysis of control and experimental B16-F1 mouse melanoma tumors. Numerous genes differentially regulated as revealed from RNA-sequencing analysis on primary B16-F1 tumors from syngeneic mice after treatment with control IgG alone, SAM, anti-PD-1, and SAM-anti-PD-1. **(A)** Venn diagrams showing significant differentially regulated genes (FDR < 0.05) in SAM vs. control group, anti-PD-1 vs. control group, and SAM+anti-PD-1 vs. control group. **(B)** MA plots of all genes differentially regulated in SAM vs. control group, anti-PD-1 vs. control group, and SAM+anti-PD-1 vs. control group. The red dots describe genes that were significantly up- or downregulated, and the black dots represent non-significant genes. **(C)** Venn diagram representing significant (FDR < 0.05) genes differentially regulated in all three groups and overlapping genes between groups. **(D)** The differentially expressed genes (DEGs) obtained from RNA-seq data were overlapped with The Melanoma Gene Database (MGDB). MGDB is a database of 422 known melanoma protein-coding genes (41). (Left) DEGs obtained from SAM+anti-PD-1 group overlapped with MGDB genes; (Right) common DEGs from each group SAM, anti-PD-1, and SAM+anti-PD-1 group and the MGDB were plotted to show common and unique genes between each treatment group.

We next determined which molecular pathways are triggered by a combination of SAM and anti-PD-1 and are possibly involved in the enhanced antitumor effects. We performed RNA sequencing analysis on primary tumors isolated from the control, SAM, anti-PD-1, and combination treated mice. Differential gene expression analysis revealed numerous genes significantly (FDR < 0.05) up- or downregulated in SAM, anti-PD-1, and the combination when compared to control as shown in Figure 3. The combination of SAM and anti-PD-1, when compared to the control group, showed a high number (887 up- and 847 downregulated) of significantly (FDR < 0.05) differentially expressed genes (DEGs) than either monotherapy. This differential regulation indicated that combination treatment simultaneously affected several pathways, which resulted in blocking tumor growth as shown in Figure 2. The pathway analysis of downregulated genes showed various pathways that were enriched in combination treatment compared to monotherapy and control (Figure 3). These repressed pathways were mainly involved in cancer, cell cycle, DNA repair, and immune system (Table 1). Various MAPK and tyrosine kinases pathways that are major oncogenic pathways involved in melanoma tumorigenesis were significantly downregulated in tumors treated with the combination of SAM+anti-PD-1 but not in SAM (except one of the MAPK pathways) and anti-PD-1 alone (Table 1) (41, 42). In contrast, pathways that were upregulated were mainly involved in mRNA processing, translation, metabolism, and transcription (Table 2).

**Table 1:** Representative key pathways downregulated as revealed from gene enrichment analysis using Consensus PathDB on downregulated genes from RNA-sequencing analysis of primary B16-F1 tumors treated with SAM+anti-PD-1 compared to Control.

	**Pathway name**	***p*-value**
Cancer	NRAGE signals death through JNK	3.49E-08
	Cell death signaling via NRAGE, NRIF and NADE	3.17E-07
	PTEN regulation	1.54E-05
	Death receptor signaling	2.80E-05
	Regulation of TP53 activity through acetylation	3.30E-05
	mTOR signaling pathway–Mus musculus (mouse)	0.000778
	ErbB signaling pathway–Mus musculus (mouse)	0.00285
	Endometrial cancer–Mus musculus (mouse)	0.011
	Colorectal cancer–Mus musculus (mouse)	0.0121
	Androgen receptor signaling pathway	0.0194
	Breast cancer–Mus musculus (mouse)	0.0225
	Hepatocellular carcinoma–Mus musculus (mouse)	0.0302
	Glioma–Mus musculus (mouse)	0.0304
	Beta-catenin phosphorylation cascade	0.0385
	Wnt signaling pathway	0.041
	Pathways in cancer–Mus musculus (mouse)	0.0435
Cell cycle	Cell cycle	9.37E-06
	Cell cycle, mitotic	0.000257
	Mitotic prometaphase	0.000265
	M phase	0.000639
	Cell cycle checkpoints	0.0113
	G2/M checkpoints	0.0378
	G2/M transition	0.0181
	Mitotic G2-G2/M phases	0.0201
DNA repair	SUMOylation of DNA damage response and repair proteins	0.00163
	Homology directed repair	0.00217
	DNA double-strand break repair	0.00652
	DNA repair	0.00793
	HDR through Single Strand Annealing (SSA)	0.0135
	HDR through Homologous Recombination (HR) or Single Strand Annealing (SSA)	0.0138
	HDR through Homologous Recombination (HRR)	0.0183
	Homologous DNA pairing and strand exchange	0.0287
Other cancer related pathways	Regulation of PTEN stability and activity	0.00123
	PIP3 activates AKT signaling	0.00179
	Signaling by TGF-beta receptor complex	0.00327
	Wnt signaling pathway NetPath	0.00604
	Regulation of TP53 activity	0.00654
	MAPK1/MAPK3 signaling	0.00847
	Phosphatidylinositol signaling system–Mus musculus (mouse)	0.00854
	Transcriptional regulation by E2F6	0.00902
	Regulation of PTEN gene transcription	0.00908
	Neurophilin interactions with VEGF and VEGFR	0.0102
	MAPK family signaling cascades	0.0115
	Antigen processing: ubiquitination & proteasome degradation	0.0115
	Proteoglycans in cancer–Mus musculus (mouse)	0.0133
	Transcriptional regulation by TP53	0.0139
	RAF/MAP kinase cascade	0.014
	EGFR1 signaling pathway	0.0177
	AMPK signaling pathway–Mus musculus (mouse)	0.0181
	RAF activation	0.0228
	3-phosphoinositide biosynthesis	0.0228
	Signaling by EGFR	0.0236
	Signaling by TGF-beta family members	0.0329
	Regulation of PTEN localization	0.0424
	EGFR transactivation by gastrin	0.0424
	MAPK signaling pathway–Mus musculus (mouse)	0.0451
	Signaling by receptor tyrosine kinases	0.0488

**Table 2:** Representative key pathways upregulated as revealed from gene enrichment analysis using Consensus PathDB on upregulated genes from RNA-sequencing analysis of primary B16-F1 tumors treated with SAM+anti-PD-1 group compared to Control.

	**Pathway name**	***p*-value**
mRNA processing	mRNA capping	7.37E-05
	mRNA processing	7.72E-15
	mRNA splicing	5.75E-13
	mRNA splicing–major pathway	2.69E-11
	mRNA splicing–minor pathway	2.82E-09
	Metabolism of RNA	5.73E-09
	Spliceosome–Mus musculus (mouse)	2.02E-14
Translation	Ribosome–Mus musculus (mouse)	6.14E-93
	Eukaryotic translation initiation	9.34E-33
	Translation initiation complex formation	1.58E-32
	Metabolism of proteins	2.54E-11
	Proteasome–Mus musculus (mouse)	1.19E-07
	Proteasome degradation	3.82E-06
	Protein export–Mus musculus (mouse)	7.37E-05
	Targeted protein degradation	1.38E-05
Metabolism	Oxidative phosphorylation–Mus musculus (mouse)	6.80E-44
	Electron transport chain	5.74E-43
	Translation	5.58E-42
	Aerobic respiration—electron donor II	6.63E-39
	Respiratory electron transport, ATP synthesis by chemiosmotic coupling, and heat production by uncoupling proteins.	3.88E-31
	NADH to cytochrome bo oxidase electron transfer	5.26E-29
	NADH to cytochrome bd oxidase electron transfer	5.26E-29
	Oxidative phosphorylation	2.89E-26
	Respiratory electron transport	1.18E-24
	The citric acid (TCA) cycle and respiratory electron transport	2.20E-23
	Oxidative stress	0.000601
Transcription	RNA polymerase–Mus musculus (mouse)	7.58E-06
	RNA polymerase I chain elongation	9.40E-05
	RNA polymerase I promoter escape	0.000119
	RNA polymerase I transcription termination	0.000148
	RNA polymerase II promoter escape	0.000294
	RNA polymerase II transcription initiation and promoter clearance	0.000344
	RNA polymerase II transcription pre-initiation and promoter opening	0.000344
	RNA polymerase II transcription initiation	0.000344
	RNA polymerase III transcription initiation from type 1 promoter	0.000601
	RNA polymerase III transcription initiation from type 3 promoter	0.000601
	Eukaryotic transcription initiation	0.000776
	RNA transport–Mus musculus (mouse)	0.000779
	RNA polymerase II transcription elongation	0.00134
	Formation of RNA Pol II elongation complex	0.00134
	RNA polymerase I transcription initiation	0.0016
	RNA polymerase III transcription initiation	0.00239
	RNA polymerase III transcription	0.00239
	RNA polymerase II pre-transcription events	0.00341
	RNA polymerase I promoter clearance	0.00857
	RNA polymerase I transcription	0.00913
	Gene silencing by RNA	0.0131
	RNA polymerase II transcription termination	0.0137
	mRNA 3,-end processing	0.0262
	RNA degradation–Mus musculus (mouse)	0.0419

Next, we overlapped our DEGs of tumors treated with SAM+anti-PD-1 (compared to control) with known melanoma cancer genes from The Melanoma Gene Database (MGDB) that has 422 melanoma-specific protein-coding genes (41) and The Cancer Genome Atlas (TCGA) (Figure 3D and Supplementary Figure 3) (42, 43). We found 28 melanoma-specific genes to be common between our data and MGDB, out of which, 18 DEGs were downregulated and 10 were upregulated with SAM+anti-PD-1 treatment. However, only one was downregulated with SAM and two with anti-PD-1 antibody (Figure 3D). We analyzed a few of the top DEGs (*NRP2, CAPN3, DMBT1, BRAF, DDIT3, PPP1R3C, NF1*) using The UCSC Xena platform (44) that has large number of RNA-seq data of normal tissue from healthy individuals (GTEx) and primary tumor and metastatic tissue data from melanoma patients (TCGA).

**Figure 4:**
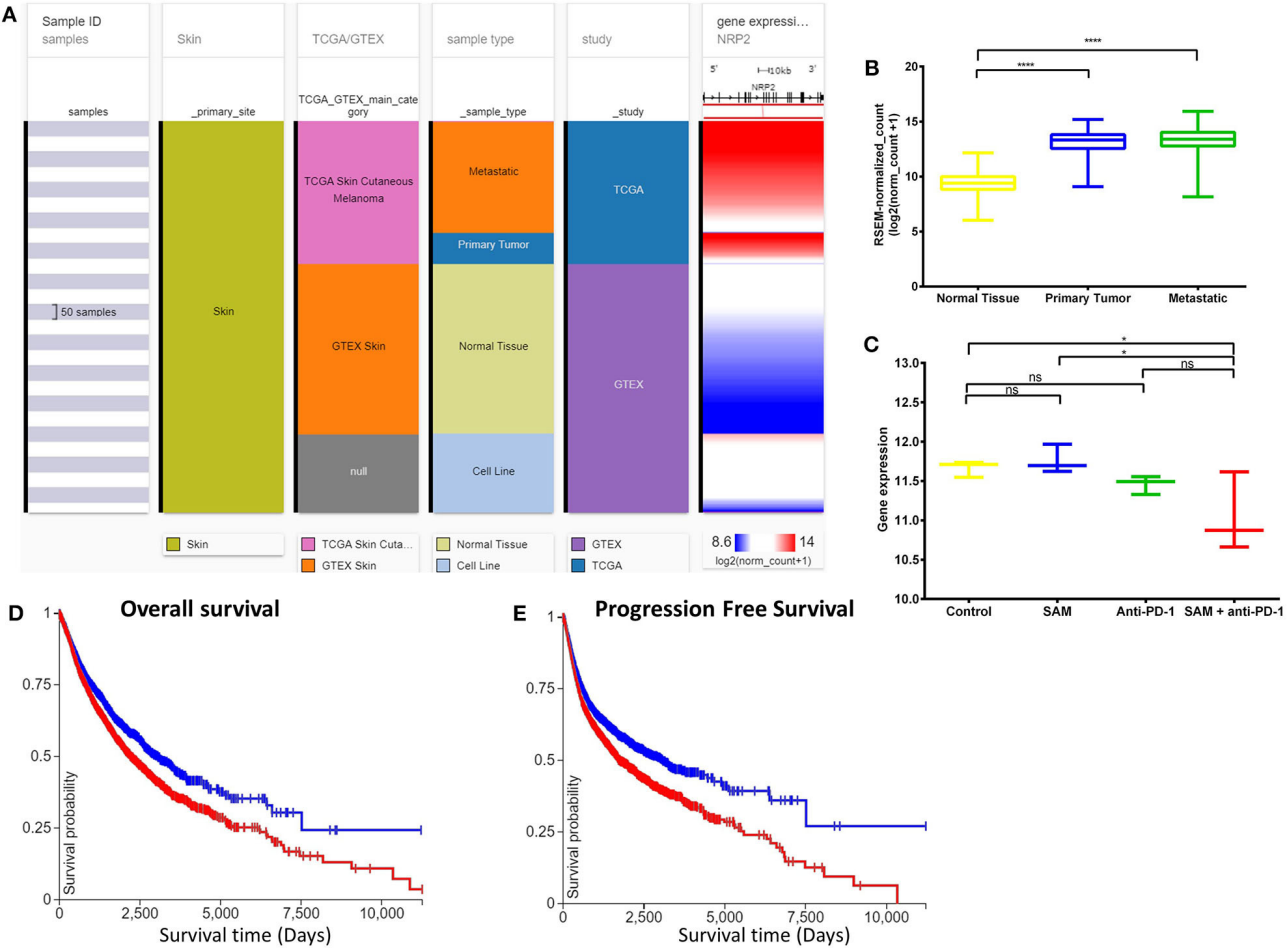
Analysis of *NRP2* gene expression in clinical public data. **(A)** Expression of *NRP2* gene in human healthy and skin cutaneous melanoma patients of GTEx and TCGA databases, respectively. The columns show various phenotypic categories applied to stratify samples according to Sample Id, Skin (true), TCGA/GTEX, sample type (normal tissue, primary tumor, metastatic tissue, or cell line), and study. The last column shows gene expression of *NRP2* of samples stratified according to the previous columns. Each row contains data from a single sample. **(B)** The expression data of *NRP2* in the normal tissue, primary tumor, and metastatic tissue samples in **(A)** have been plotted in a box-plot graph (*n* = 1,024 samples). Expression values are in RSEM (RNA-Seq by expectation maximization). **(C)** The expression data of *Nrp2* from RNA-sequencing of the primary B16 tumors after treatment with SAM, Anti-PD-1, and combination in this study (*n* = 12; 3/group). Expression values are DeSEq2 normalized counts. **(D,E)** Overall survival and progression-free survival Kaplan-Meier curves of *NRP2* from RNA-seq of GTEx and TCGA databases; X-axis: survival time (days); Y-axis: survival probability. **(D)** Low (blue) *n* = 4,504; High (red) *n* = 5,930; *P* = ****. **(E)** Low (blue) *n* = 4,346; High (red) *n* = 5,926; *P* = ****. Statistical significance was obtained by ANOVA in GraphPad prism and are represented by asterisks (ns, not significant; *P < 0.05 and ****P < 0.0001). All the data and figures, except **(C)**, were generated using The UCSC Xena platform (44).

Neuropilins (NRPs) function as coreceptors of the VEGF family and plexins and are involved in promoting angiogenesis and in axonal guidance, respectively (45, 46). NRP2 was recently found to be an oncogene involved in accelerating melanoma tumor growth and progression *in vivo* (45, 46). *NRP2* showed significantly high expression in the primary tumors and metastatic tissues of the melanoma patient samples although normal tissues had low expression (Figures 4A,B). In addition, *NRP2* had the highest expression in melanoma TCGA data compared to all other cancers in the TCGA Pan-Cancer Atlas (Supplementary Figure 4). Interestingly, the tumor-bearing mice treated with the combination of SAM+anti-PD-1 had the lowest expression of *Nrp2* compared to other groups (Figure 4C). Tumors treated with SAM+anti-PD-1 showed significant downregulation of *Nrp2* compared to control (*p* < 0.05) although *Nrp2* expression in tumors treated with SAM and anti-PD-1 alone were not found to be significantly downregulated in RNA-seq data. Downregulation of *Nrp2* expression in SAM+anti-PD-1 treated tumors (*n* = 4 tumors/group) was further validated using RT-qPCR (Supplementary Figure 5). Moreover, high expression of *NRP2* was found to have significantly low overall survival and progression-free survival rates (*p* < 0.0001) in melanoma patients (Figures 4D,E).

**Figure 5:**
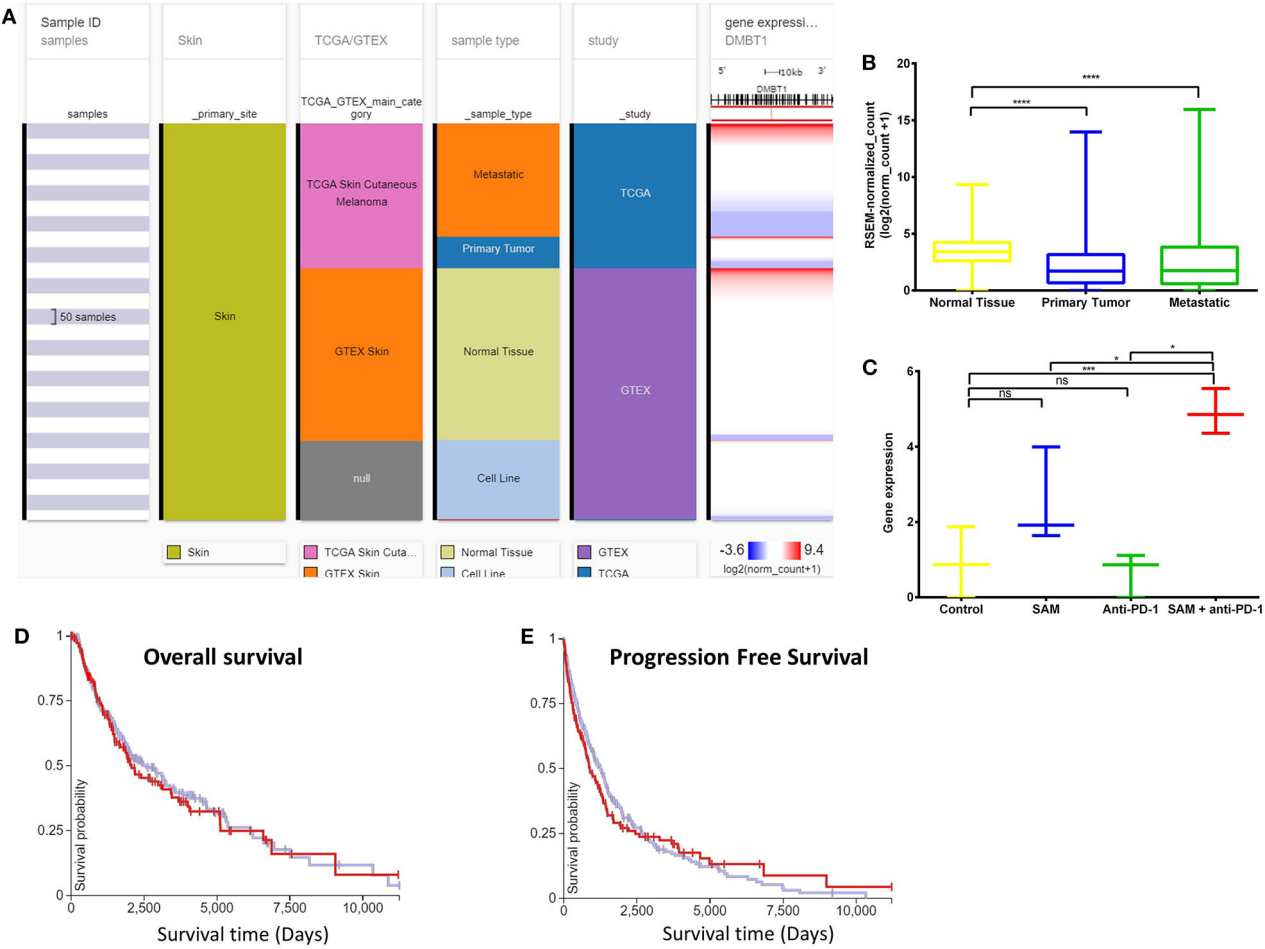
Analysis of *DMBT1* gene expression in clinical public data. **(A)** Expression of *DMBT1* gene in human healthy and skin cutaneous melanoma patients of GTEx and TCGA databases, respectively. The columns show various phenotypic categories applied to stratify samples according to Sample Id, Skin (true), TCGA/GTEX, sample type (normal tissue, primary tumor, metastatic tissue, or cell line), and study. The last column shows gene expression of *DMBT1* of samples stratified according to the previous columns. Each row contains data from a single sample. **(B)** The expression data of *DMBT1* in the normal tissue, primary tumor, and metastatic tissue samples in (A) has been plotted in a box-plot graph (*n* = 1,024 samples). Expression values are in RSEM (RNA-Seq by expectation maximization). **(C)** The expression data of *Dmbt1* from RNA-sequencing of the primary B16 tumors after treatment with SAM, Anti-PD-1, and combination in this study (*n* = 12; 3/group). Expression values are DeSEq2 normalized counts. **(D,E)** Overall survival and progression-free survival Kaplan–Meier curves of *DMBT1* from RNA-seq of GTEx and TCGA databases; X-axis: survival time (days); Y-axis: survival probability. **(D)** Low (blue) *n* = 302; High (red) *n* = 153; *P* = ns **(E)** Low (blue) *n* = 302; High (red) *n* = 154; *P* = ns. Statistical significance was obtained by ANOVA in GraphPad prism and are represented by asterisks (ns, not significant; *P < 0.05, ***P < 0.001, and ****P < 0.0001). All the data and figures, except **(C)**, were generated using The UCSC Xena platform (44).

Deleted in malignant brain tumors 1 (*DMBT1*) has been reported to be a tumor suppressor gene (TSG) in brain (medulloblastoma, GBM), lung, and gastrointestinal tumors based on homozygous deletions, lack of expression, its instability in cancer, and having key roles in immune defense and epithelial differentiation (47). *DMBT1* showed significantly low expression in the primary tumors and metastatic tissues of the melanoma patient samples although normal tissue had high expression (Figures 5A,B). In addition, *DMBT1* had one of the lowest expressions in melanoma TCGA data compared to all other cancers in the TCGA Pan-Cancer Atlas (Supplementary Figure 6). The tumor-bearing mice that were treated with the combination of both SAM+anti-PD-1 had the significantly highest expression of *Dmbt1* compared to control, SAM alone, and anti-PD-1 alone (Figure 5C). Tumors treated with SAM+anti-PD-1 showed significant upregulation of *Dmbt1* compared to control (*p* < 0.001) although *Dmbt1* expression in tumors treated with SAM and anti-PD-1 alone were not found to be significantly upregulated in RNA-seq data. Upregulation of *Dmbt1* expression in SAM+anti-PD-1 treated tumors (*n* = 4 tumors/group) was further validated using RT-qPCR (Supplementary Figure 5). *DMBT1* was not found to have a good prognostic value in melanoma (Figures 5D,E), but high expression of *DMBT1* was favorable in endometrial cancer (43). *Braf* and *Nf1*, known melanoma driver genes (42), were found to be significantly downregulated in tumors treated with SAM+anti-PD-1 compared to control. *BRAF* and examples of a few other genes are shown in Supplementary Figures 7–14. These data may indicate that the combination of SAM+anti-PD-1 therapy reversed the expression of some of the aberrantly expressed genes in melanoma, which might be underpinning its therapeutic effect against melanoma tumors in mice.

Next, we validated the highest significantly down- (*Myh2, Mybh, Sypl2, Xirp1, Mybpc1*) and up- (*Fcgbp, Areg*) regulated genes, including the melanoma-specific genes (*Dmnt1* and *Nrp2*) identified by RNA sequencing following treatment with SAM+anti-PD-1 by RT-qPCR. These genes were similarly up-/downregulated in primary tumoral RNA of mice treated with SAM+anti-PD-1 (Supplementary Figure 5).

### Beneficial Effect of SAM and Anti-PD-1 Combinatorial Therapy on Anticancer Immune Response

**Figure 6:**
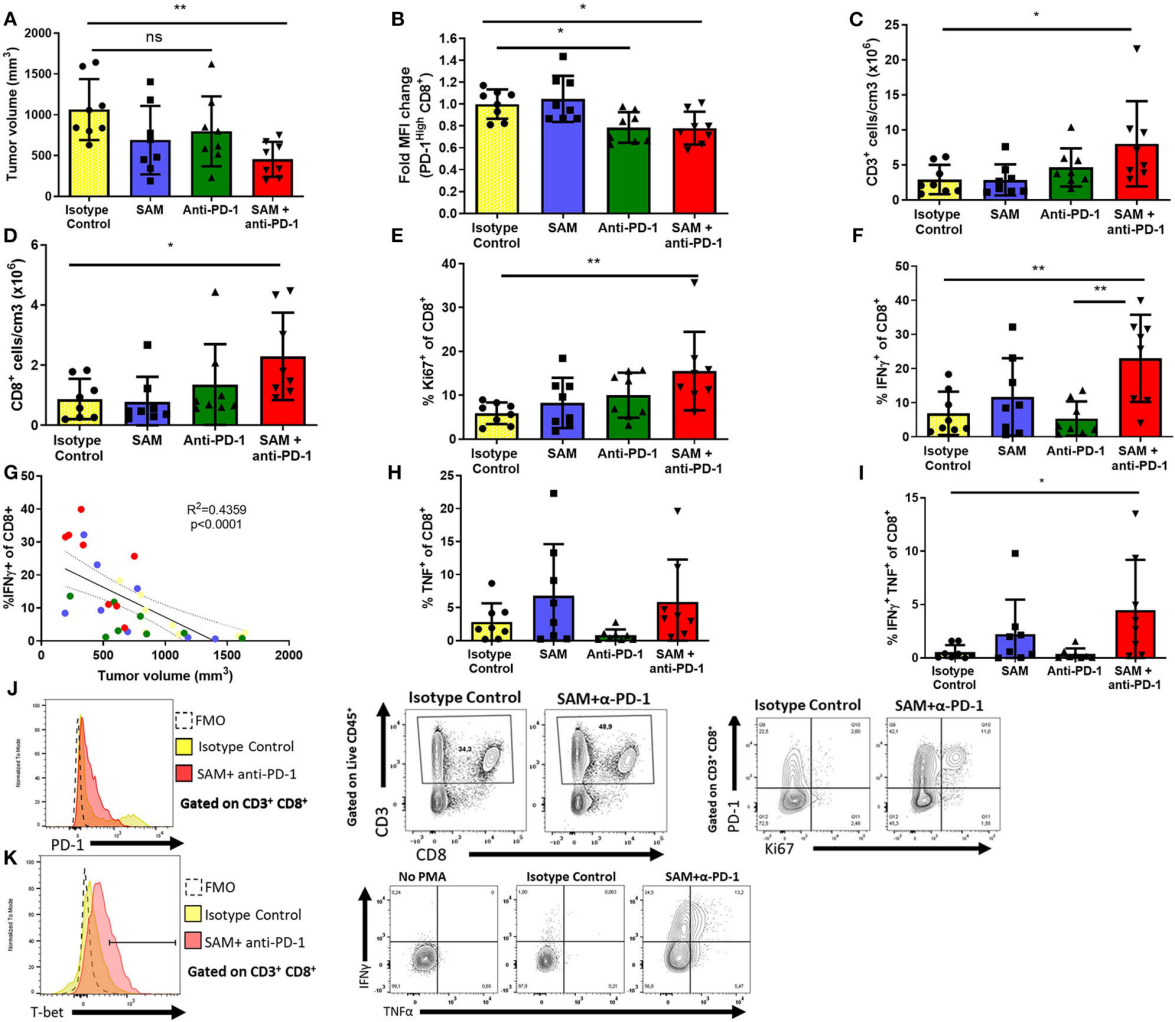
Effect of SAM, anti-PD-1, and SAM+anti-PD-1 on immune responses in the tumor microenvironment as determined by tumor immune-phenotyping using flow cytometry. B16-F1 tumor-inoculated mice were treated with control IgG alone (control), SAM, anti-PD-1 antibody, and SAM+anti-PD-1 antibody, and mice were sacrificed at day 14 and subjected to immune-phenotyping as described in Materials and Methods. **(A)** Tumor volume was measured at day 14, and results are representative of mean ± SEM (*n* = 8 mice per group from two independent experiments). **(B)** Fold change mean fluorescence intensity (MFI) of CD8+ T cells expressing high levels of PD-1. **(C,D)** Number of CD3^+^ and CD8^+^ cells per cm^3^ of tumor tissue, respectively. **(E,F,H,I)** Percentage of Ki67^+^, IFN-γ^+^, TNFα^+^, IFN-γ^+^, and TNFα^+^ T cells, respectively, in all the groups tested. **(G)** Correlation analysis of percentage (%) of IFN-γ^+^ CD8^+^ T cells against tumor volume (mm^3^) of all mice in the four groups tested. **(J)** Representative flow plots for expression of CD3, CD8 (middle), PD-1 (left), and Ki67 (right) in tumor-infiltrating lymphocytes. Fluorescence Minus One (FMO) was used as technical control to determine gates. **(K)** Representative flow plots for expression of T-bet, IFNγ, and TNFα after PMA/Ionomycin stimulation. No PMA stimulation is shown as a biological control. Statistical significance was obtained by ANOVA in GraphPad prism and are represented by asterisks (*P < 0.05 and **P < 0.01).

We carried out immuno-phenotyping of infiltrating cells from primary tumors of the control group and mice treated with SAM, anti-PD-1 antibody, and SAM+anti-PD-1 (Figure 6). Here, we opted for a suboptimal administration scheme of anti-PD-1 to parse out the additive effects of combination therapy. To confirm the immune effect of anti-PD-1 treatment, we assessed the level of expression of PD-1 on CD8^+^ tumor infiltrating lymphocytes (TILs) at the end point by flow cytometry. In both groups having received anti-PD-1, we observed a 20% reduction of PD-1 mean fluorescence intensity (MFI) among PD-1^High^ CD8^+^ T cells (Figures 6B,J). In this experiment, only the SAM+anti-PD-1 arm displayed a significant reduction in tumor volume at the end point (Figure 6A). Accordingly, the density of tumor-infiltrating T cells (CD45^+^ CD3^+^) and CD8^+^ T cells, measured as number of cells/cm^3^ of tumor, was significantly increased in the combination therapy group (Figures 6C,D). We observed a corresponding increase in the proportion of CD8^+^ T cells in the tumor-draining lymph node, suggesting increased expansion and/or recruitment of CD8^+^ cells to the tumor (Supplementary Figures 15A,B). Furthermore, the proliferation of CD8^+^ T cells, measured by expression of the mitotic marker Ki67, was significantly increased in the SAM+anti-PD-1 group (Figure 6E), and anti-PD-1 also restored the proliferative capacity of PD-1^+^ TILs (Figure 6J).

As CD8^+^ T cells are known to be potent effectors of antitumor responses, we then sought to characterize their cytokine-production capabilities. Despite a high level of variability in the tumors of the control group, we observed a significant increase in the percentage of CD8^+^ T cells secreting IFNγ after polyclonal PMA/Ionomycin stimulation in the combination group (mean = 23.0 ± 12.7%), compared to the control (6.88 ± 6.35%) and anti-PD-1 monotherapy group (5.33 ± 5.02%) (Figures 6F,K). This high level of variability was explained by the heterogeneity of tumor sizes at the end point. Indeed, there was a strong negative correlation between the frequency of CD8^+^ T cells secreting IFNγ and tumor size at the end point (*r*^2^ = 0.436, *p* < 0.0001), suggesting that IFNγ^+^ CD8^+^ cells confer protective antitumor immunity in our model (Figure 6G and Supplementary Figure 15C). Furthermore, despite not observing a significant difference in the proportion of CD8^+^ T cells secreting TNFα, combination therapy readily induced a population of IFNγ^+^ TNFα^+^ CD8^+^ cells that was mostly absent in all other treatment arms (Figures 6H,I). Finally, CD8^+^ T cells from the combination group upregulated T-bet expression in CD8^+^ cells (Supplementary Figure 15D). Notably, this overall increase in proliferation and effector functions was not observed in conventional CD4^+^ T cells (CD4^+^Foxp3^−^) (Supplementary Figures 15E–H).

We assessed the frequency of myeloid cell subsets as well as their level of PD-L1 expression (Supplementary Figures 16–18). We did not observe any significant difference in the frequency of macrophages (CD11b+ F4/80^High^), dendritic cells (CD11c^+^), neutrophils and granulocytic-myeloid-derived suppressive cells (MDSCs, Ly6G^+^ Ly6C^Int^), monocytes and monocytic MDSCs (CD11b^+^ Ly6C^+^ F480^int^) (Supplementary Figures 16–18). However, we observed an increase in the frequency of PD-L1^+^ macrophages, monocytes, and M-MDSCs and CD11b^+^ dendritic cells (Supplementary Figure 16G–L). Furthermore, the level of expression of PD-L1, measured by MFI, was increased in three out of four mice in the combination group. PD-L1 expression is known to be inducible by IFNγ, and paradoxically, high levels of PD-L1 expression have been proposed as a predictive marker of the response to anti-PD-1 (1). Taken together, these data show that treatment with SAM potentialized the efficacy of anti-PD-1 and increased antitumor immunity through a specific activation and proliferation of CD8^+^ T cells, recapitulating known hallmarks of response to treatment.

## Discussion

Immune checkpoint inhibitors (ICPi) received FDA approval as early as 2011 for the treatment of advanced melanoma (1, 18, 20). However, despite melanoma being the solid tumor type most responsive to the anti-PD-1 monoclonal antibody, overall response rates are estimated around 30–33%, indicating that a considerable number of patients do not experience a reduction in tumor burden, resulting in high morbidity and mortality (1, 18, 20–22). The immunological basis of treatment failure is a very actively researched topic. Nevertheless, considering the tremendous clinical improvements experienced by high responder patients, there is a need for therapeutic strategies to potentialize the effect of anti-PD-1 and strengthen antitumor immunity. Here, we show that the combination of an approved nutraceutical, the epigenetic modulator SAM, with an anti-PD-1 antibody displayed strong anticancer effects against B16 cells, the most commonly used preclinical syngeneic mouse model of advanced melanoma. Furthermore, using a suboptimal administration scheme of anti-PD-1 in which the tumor burden is not reduced by monotherapy, we provide evidence that coadministration of SAM is sufficient to potentialize the effect of anti-PD-1 and induce a strong antitumor immune response.

Previous studies have demonstrated that global and target gene–specific hypomethylation are present in the cancer epigenome, which plays a crucial role in the initiation and progression of cancer (4). Furthermore, there is insufficient SAM available in the tumor microenvironment (48). SAM treatment results in significant antitumor effects in breast, osteosarcoma, prostate, hepatocellular, gastric, colon, and other cancers (6–10). Here, we show the significant anticancer effect of SAM as monotherapy in a model of advanced melanoma that is at least as effective as anti-PD-1 treatment. The fact that an approved nutraceutical agent, SAM, with a good safety profile, shows potentiating effects on anti-PD-1 in a model resistant to immunotherapy should encourage translation of these findings to the clinic.

Human anti-PD-1 antibodies (nivolumab and pembrolizumab) are currently recommended as the first line of treatment in advanced melanoma and are FDA-approved for several other cancer indications. The PD-1/PD-L1 signaling axis dampens TCR and CD28 signaling in T cells and is hijacked by PD-L1 expressing tumor cells to deactivate antitumor responses (1, 18, 20). However, PD-L1 has been extensively reported to have intrinsic signaling in various cancer cell types, which promotes cancer initiation, metastasis, development, resistance to therapy, enhances cancer cell survival, regulates stress responses, and confers resistance toward pro-apoptotic stimuli (37, 38). Hence, we investigated the consequences of blocking the PD-1/PD-L1 pathway *in vitro* using B16-F1 cells. To induce PD-L1 signaling, we first added rPD-1 in the medium and then blocked the PD-1/PD-L1 pathway with anti-PD-L1 antibody (26). We didn't use anti-PD-1 antibody *in vitro* as the monoclonal antibody would bind and neutralize rPD-1 directly. The anticancer effect of anti-PD-L1 on B16-F1 cells was low, which is consistent with the previously published literature showing that the protective effect of this ICPi is mainly through the enhancement of the immune response (1, 17–20).

To study the impact of SAM on tumor control *in vivo*, we used a murine anti-PD-1 antibody as a comparator because it is the standard of care for human advanced melanoma patients. Having first shown that SAM had similar protection to anti-PD-1 in immunocompetent mice, we then opted for a suboptimal anti-PD-1 administration scheme to model for treatment failure and demonstrate the superior effect of SAM with anti-PD-1. In this setting, anti-PD-1 monotherapy decreased the level of PD-1 expression on CD4^+^ and CD8^+^ T cells but failed to increase CD8^+^ infiltration and effector functions in the tumor microenvironment. However, coadministration of SAM was sufficient to restore protective immunity. Mice in the combination group recapitulated known hallmarks of successive response to PD-1 blockade, namely increased infiltration, proliferation, and secretion of IFNγ and expression of T-bet by CD8^+^ T cells. Polyfunctional CD8^+^ T cells secreting both IFNγ and TNFα are highly active effector CD8^+^ T cells that are associated with improved antitumor immunity in preclinical mouse models and in patients and are considered to be potent mediators of antitumor activity (49). The combination therapy of SAM with anti-PD-1 antibody induced a higher population of polyfunctional CD8^+^ T cells.

Despite its efficacy in the clinic, it is well-established that the protective effect of murine anti-PD-1 monotherapy is less potent in the B16-F1 model (32, 50). Indeed, this model is considered very aggressive and poorly immunogenic with low levels of MHC I expression in these cells (40). Also, early preclinical models that demonstrated the protective effect of anti-PD-1 used vaccination with irradiated B16 melanoma cells as a combinatory approach to elicit protection (51). Furthermore, other reports show no protective effect of monotherapy in models of quickly progressing B16-F1 mouse melanoma tumors through lack of clonal expansion and effector functions of antigen-specific CD8^+^ T cells (26, 52–55). In clear contrast to anti-PD-1 monotherapy, treatment with SAM+anti-PD-1 showed significant reduction in tumor growth and enhanced anticancer immunity even in a setting with fewer injections of anti-PD-1, where monotherapy alone fails to induce protection. Our data also shows that SAM not only complements the anticancer effect by reducing oncogenic gene expression, as reported herein and previously using microarray and RNA-seq analysis, but also enhances the anticancer immunity alongside anti-PD-1 (5–12, 56). Our immunophenotyping data is consistent with the previously published literature that shows SAM could potentially increase activation and proliferation of T cells, which was observed in combination with anti-PD-1 (13–16). The fact that SAM can dramatically enhance suboptimal activity of ICPi points to the possibility that it might be possible to achieve effective antitumor activity with a lower frequency of ICPi dose, thus reducing its toxicity and adverse effects.

Another objective of the current study was to determine the molecular pathways triggered by SAM, anti-PD-1, and SAM+anti-PD-1. RNA-sequencing data showed that SAM (compared to control) caused downregulation of 57 genes and upregulation of only two genes. This is consistent with previously published literature that SAM-mediated promoter hypermethylation would result in greater gene silencing (6–10, 12). Compared to the effect of SAM on DEGs, SAM+anti-PD-1 had very high number of up- (887) and downregulated (847) genes. When examining common DEGs between SAM, anti-PD-1, and SAM+anti-PD-1, it appeared that many DEGs (1,438) in the combination treatment did not overlap with DEGs triggered by either SAM or anti-PD-1 monotherapy. This implies that the major reduction in tumor growth shown by the SAM+anti-PD-1 treatment is associated with a larger pool of genes that are involved in a diverse array of molecular pathways, including downregulation of key tumorigenesis pathways of melanoma, MAPK, and tyrosine kinase–related pathways, which could not be inhibited by the monotherapy treatment. Moreover, upon deeper analysis, it was observed that the combination treatment of SAM+anti-PD-1 acted on a group of specific genes that are aberrantly expressed in melanoma tumors, which might underlie the therapeutic effects. This molecular analysis supports the conclusion that the combination of SAM and anti-PD-1 is significantly more active than the monotherapy because it launches molecular pathways that could not be triggered by either agent on its own.

A limitation of preclinical models of melanoma is their high aggressiveness with the engraftment of a large number of tumorigenic cells not recapitulating the natural course of disease progression. In untreated mice, most tumors reach a critical volume within 16 days of tumor engraftment, limiting the ability to determine long-term effects of treatment regimens. However, even with this short-term aggressive melanoma model, SAM delayed tumor growth, and the combination of SAM with anti-PD-1 had a superior protective effect and restored CD8^+^ T cell proliferation and effector functions within the tumor microenvironment. Furthermore, the combination of SAM+anti-PD-1 showed the highest tumor volume and weight reduction (69 and 71%, respectively) at day 16. Thus, future studies evaluating the effect of SAM+anti-PD-1 in a less aggressive model of melanoma and other common cancers is warranted. This study did not evaluate the adverse effects of SAM and anti-PD-1 treatment on mice extensively. However, we did not observe a significant change in the mice body weight between each group. Moreover, immune-related adverse events upon PD-1 blockade, such as reported in pharmacovigilance data, have never been described in the B16 preclinical model of melanoma. Furthermore, SAM has an excellent safety profile that warranted its licensing as a nutraceutical agent, and its anticancer effects have been shown to be selective of tumor cells without affecting normal epithelial cells (5, 6, 10). Therefore, we hypothesize that the combination of SAM with anti-PD-1 will have a similar safety profile to immunotherapy alone. However, preclinical toxicity studies are necessary to assess the safety of this treatment regimen.

In summary, this is the first evidence for the antimelanoma effects of a methylating agent such as SAM. Furthermore, adjuvantation of anti-PD-1 with SAM was sufficient to reactivate an exhausted antitumor immune response. The major advantage of this approach is that both ICPi (such as anti-PD-1) and SAM are approved agents with long-term safety profiles. This should help accelerate its clinical translation through the initiation of clinical trials in patients with melanoma and other common cancers to reduce cancer associated morbidity and mortality.

## Data Availability Statement

All relevant data generated or analyzed are available within the main article and the Supplementary Material. The raw data of this study can be provided upon request. The RNA-seq raw data has been deposited in Sequence Read Archive (SRA) database with the accession number, PRJNA613336.

## Ethics Statement

The animal study was reviewed and approved by The McGill University Animal Care Committee.

## Author Contributions

SR, MS, and AM conceived the study and experimental design. AM and AA carried out various experimental procedures, and immunophenotyping was done by AM, AA, NM, and MA, and data analysis was carried out by AM and MA. Manuscript was written by AM, SR, MS, MA, and CP. All authors read and approved the final manuscript.

## Conflict of Interest

MS is the founder of HKG Epitherapeutics and Montreal EpiTerapia. The remaining authors declare that the research was conducted in the absence of any commercial or financial relationships that could be construed as a potential conflict of interest.

## Abbreviations

SAM/SAMe: s-adenosylmethionine
PD-1: Programmed cell death 1
PD-L1: Programmed death ligand 1
ICPi: Immune checkpoint inhibitors
r-PD-1: Recombinant PD-1
DEGs: Differentially expressed genes
MAPK: Mitogen-activated protein kinase
CD: Cluster of differentiation
RT-qPCR: reverse transcriptase quantitative real-time PCR
PMA: Phorbol 12-myristate 13-acetate
MFI: Mean fluorescence intensity
FMO: Fluorescence Minus One
*DMBT1*: Deleted in malignant brain tumors 1
*NRP2*: Neuropilin 2
TSG: Tumor suppressor gene
TILs: Tumor infiltrating lymphocytes
IFNγ: Interferon-gamma
TNFα: Tumor necrosis factor alpha
TCR: T cell receptor
MHC I: Major histocompatibility complex.
