# Supplementary material for: Enhanced Anticancer Effect of a Combination of S-adenosylmethionine (SAM) and Immune Checkpoint Inhibitor (ICPi) in a Syngeneic Mouse Model of Advanced Melanoma
Source: Front Oncol. 2020 Sep 2;10:1361. doi: 10.3389/fonc.2020.01361 (PMC7492272; doi:10.3389/fonc.2020.01361)
Supplement: Supplementary file 1 [file Data_Sheet_1.PDF]

# *Supplementary Material*

## 1 Supplementary Figures

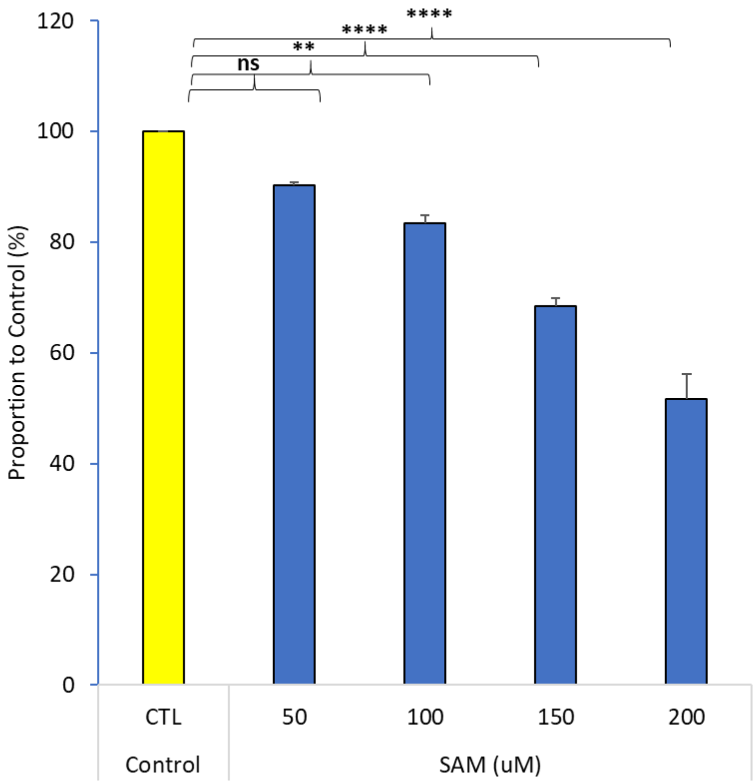


**Supplementary Figure 1: SAM dose-dependent effect on B16 cells proliferation.** B16 cells ($2x10^4$ cells) were seeded in 6-well plates. Proliferation is presented as the percentage of proportion to the Control ± SEM. Statistical significance was obtained by two-tailed T-test in Excel and are represented by asterisks (**P < 0.05; **P < 0.01, ***P < 0.001 and ****P < 0.0001*).

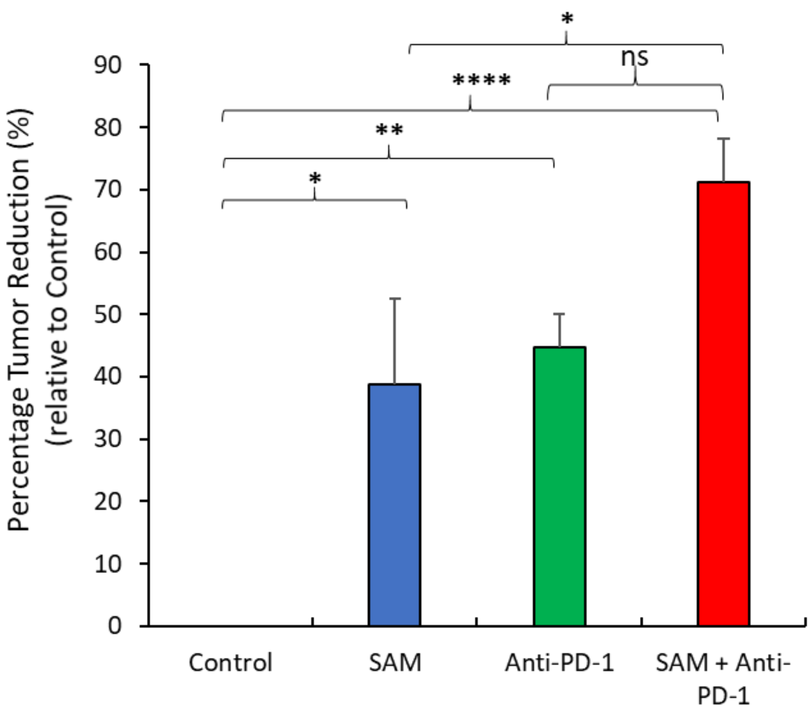


**Supplementary Figure 2: Anti-tumor effect of SAM and anti-PD-1 antibody in a syngeneic mouse B16-F1 melanoma model *in vivo*.** Percentage (%) of tumor weight reduction in each group relative to control at day 16. Results are representative of mean ± SEM of at least 8 mice per group. Statistical significance was obtained by ANOVA in GraphPad prism and are represented by asterisks (**P < 0.05; **P < 0.01, ***P < 0.001* and *****P < 0.0001*).

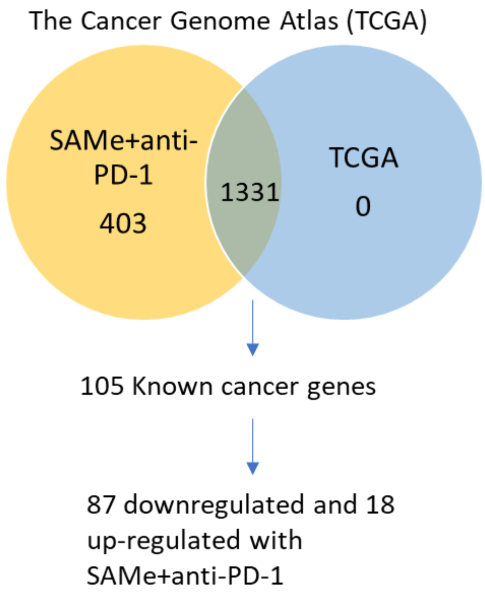


**Supplementary Figure 3: The differentially expressed genes (DEGs) obtained from RNA-seq data of SAMe+anti-PD-1 group were overlapped with TCGA database of melanoma genes.**

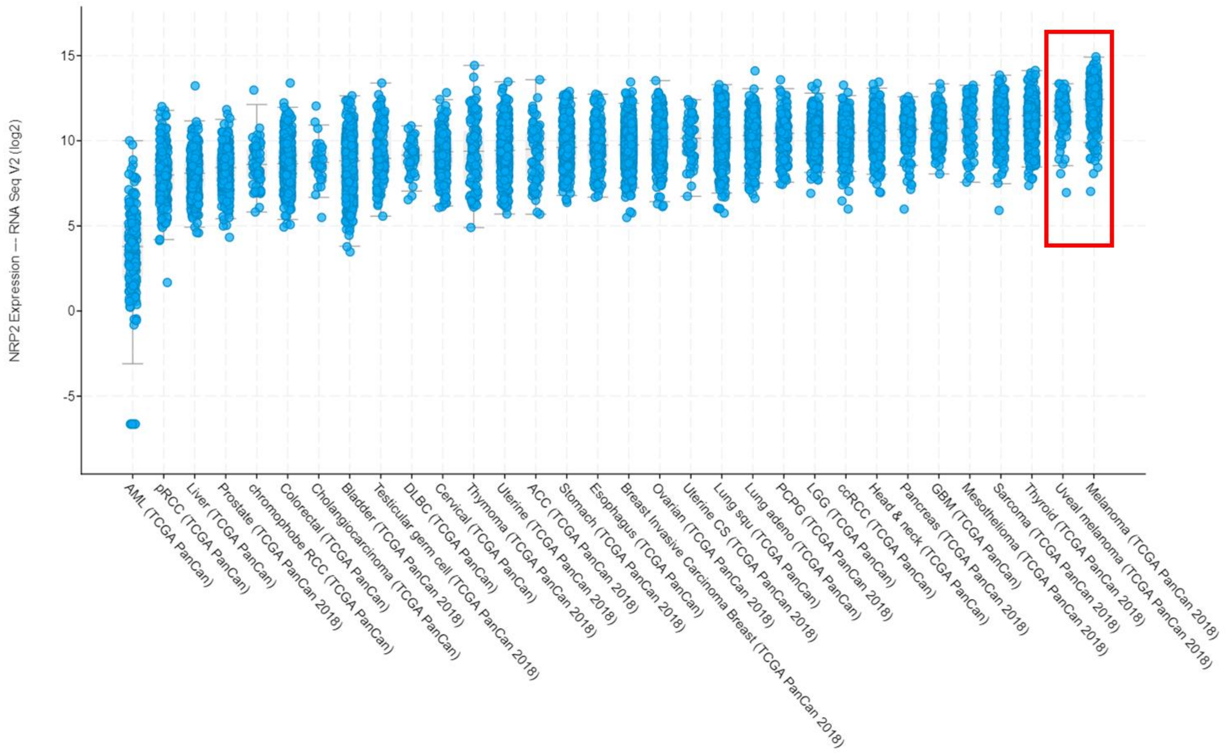


**Supplementary Figure 4: Expression of *NRP2* in the TCGA Pan-Cancer Atlas.** RNA Seq V2 (log2) median expression is plotted against each tumor type. The red box shows melanoma. Total number of samples are 10967 samples.

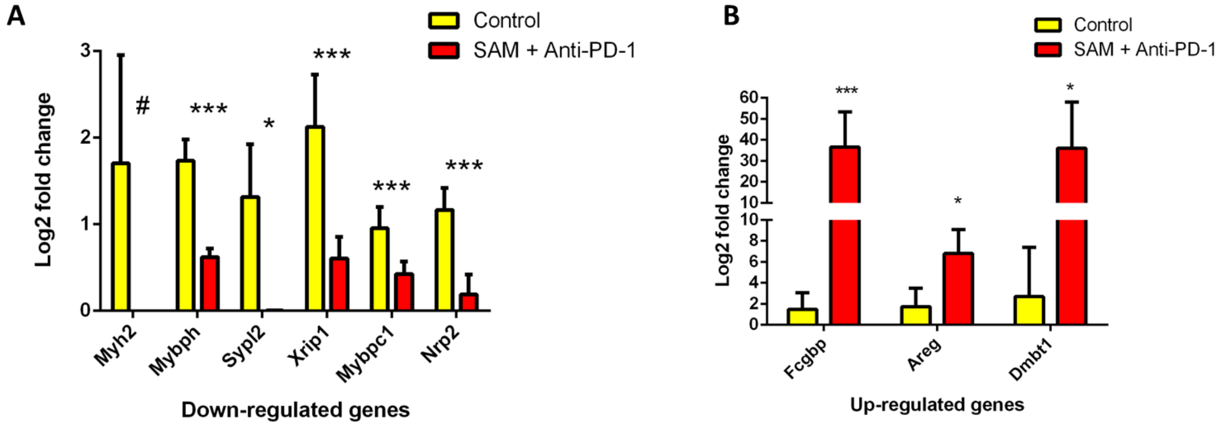


**Supplementary Figure 5: Validation of top up and down regulated genes obtained from RNA-sequencing data of control and experimental B16-F1 mouse melanoma tumors.** Top up- and down- regulated genes obtained from RNA sequencing analysis of SAMe+anti-PD-1 compared to control animals were validated by RT-qPCR. Results are mean of at least 2 independent experiments. Significant differences were determined using T-test and are represented

by asterisks (**P* < *0.05*; ***P* < *0.01*, and ****P* < *0.001*). # shows *Myh2* gene expression in the tumors of SAMe+anti-PD-1 group was undetectable.

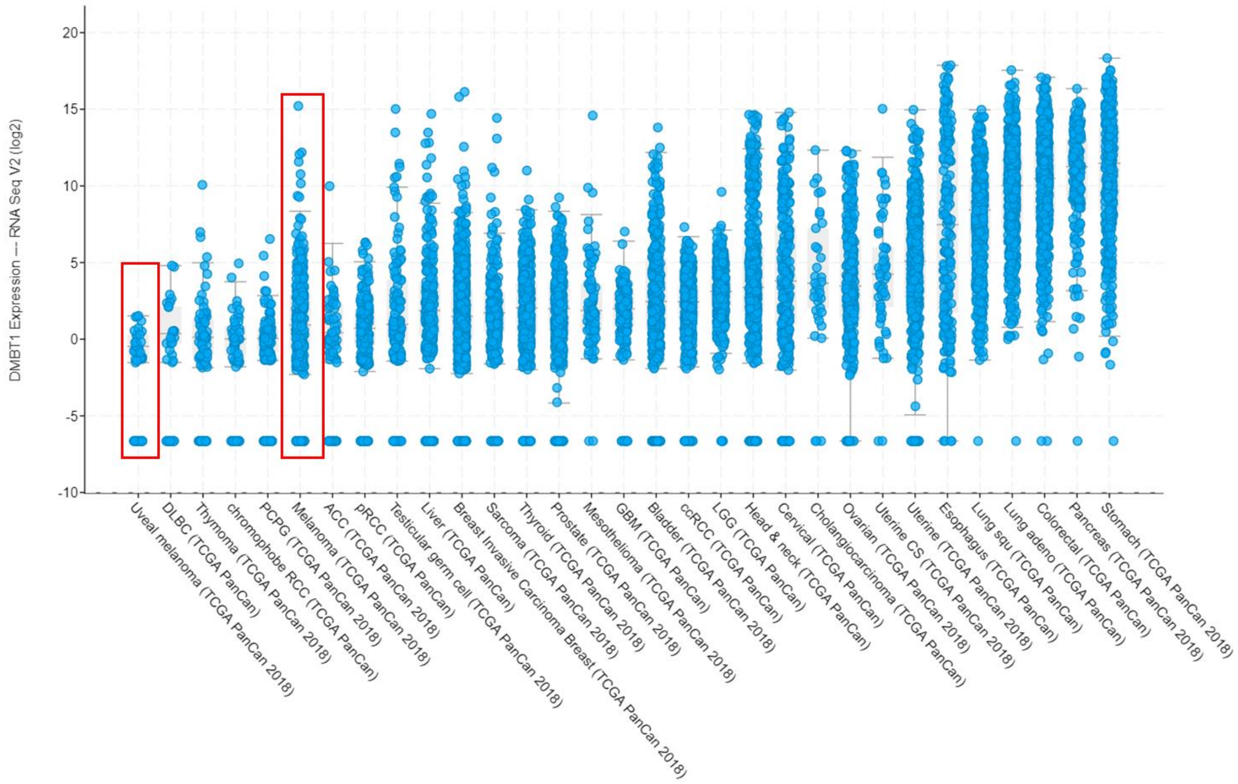


**Supplementary Figure 6: Expression of *DMBT1* in the TCGA Pan-Cancer Atlas.** RNA Seq V2 (log2) median expression is plotted against each tumor type. The red box shows melanoma. Total number of samples are 10967 samples.

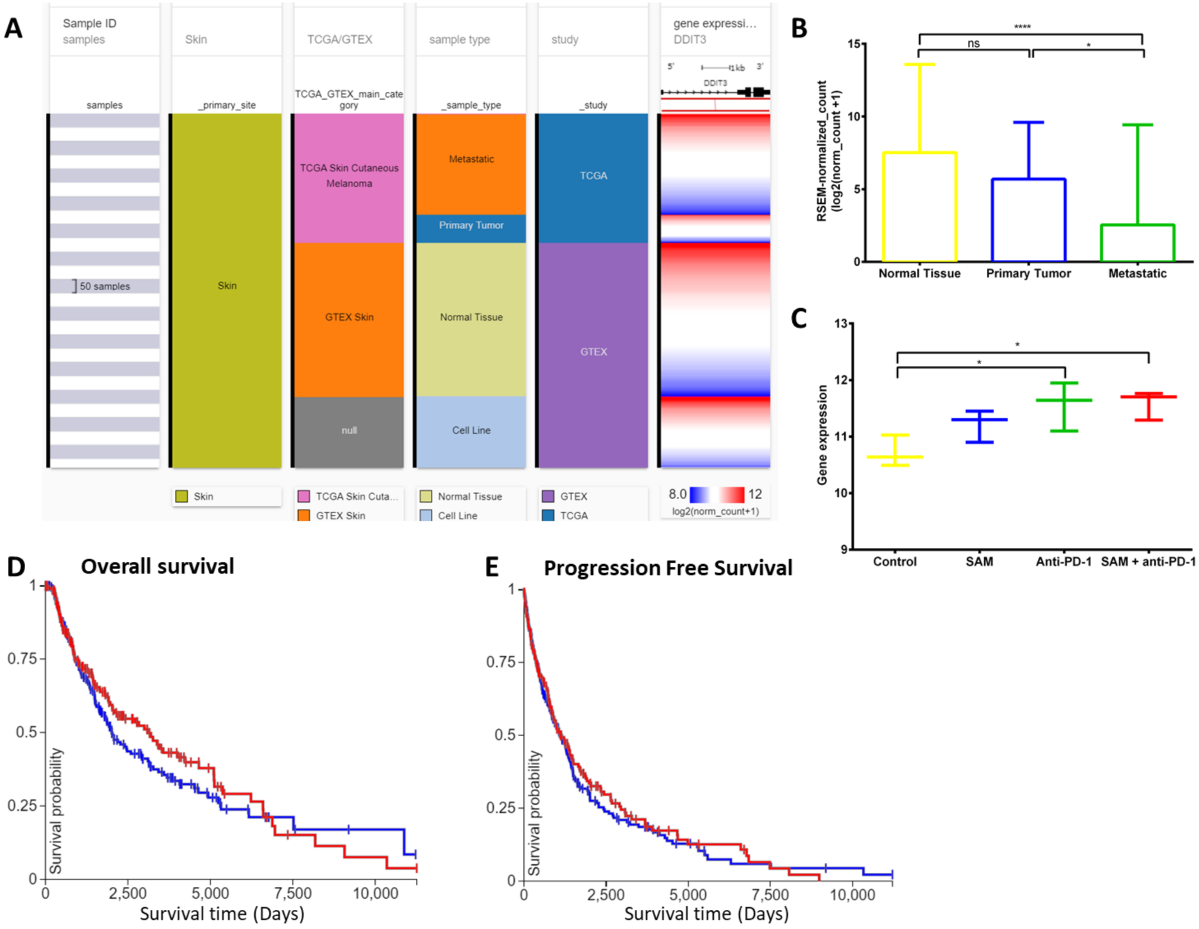


**Supplementary Figure 7: Clinical data analysis of *DDIT3* gene identified from overlapping MGDB with RNA-seq data.** (A) Expression of *DDIT3* gene in human healthy and skin cutaneous melanoma patients of GTEx and TCGA databases respectively. The columns show various phenotypic categories applied in order to stratify samples according to Sample Id, Skin (true), TCGA/GTEX, sample type (normal tissue, primary tumor, metastatic tissue or cell line) and study. The last column shows gene expression of *DDIT3* of samples stratified according to the previous columns. Each row contains data from a single sample. (B) The expression data of *DDIT3* in the normal tissue, primary tumor, metastatic tissue samples in (A) has been plotted in a Box-plot graph (n=1024 samples). Expression values are in RSEM (RNA-Seq by Expectation Maximization). (C) The expression data of *Ddit3* from RNA-sequencing of the primary B16 tumors after treatment with SAM, Anti-PD-1 and combination in this study (n =12; 3/group). Expression values are DeSEq2 normalized counts; X-axis: survival time (days); Y-axis: survival probability. (D-E) Overall survival and progression-free survival Kaplan Meier curves of *DDIT3* from RNA-seq of GTEx and TCGA databases. (D) Low (blue) n= 247; High (red) n = 208. (E) Low (blue) n= 248; High (red) n = 208. Statistical significance was obtained by ANOVA in GraphPad prism and are represented by asterisks (**P < 0.05; **P < 0.01, ***P < 0.001 and ****P < 0.0001*). All the data and Supplementary Figure s, except (C), were generated using The UCSC Xena platform.

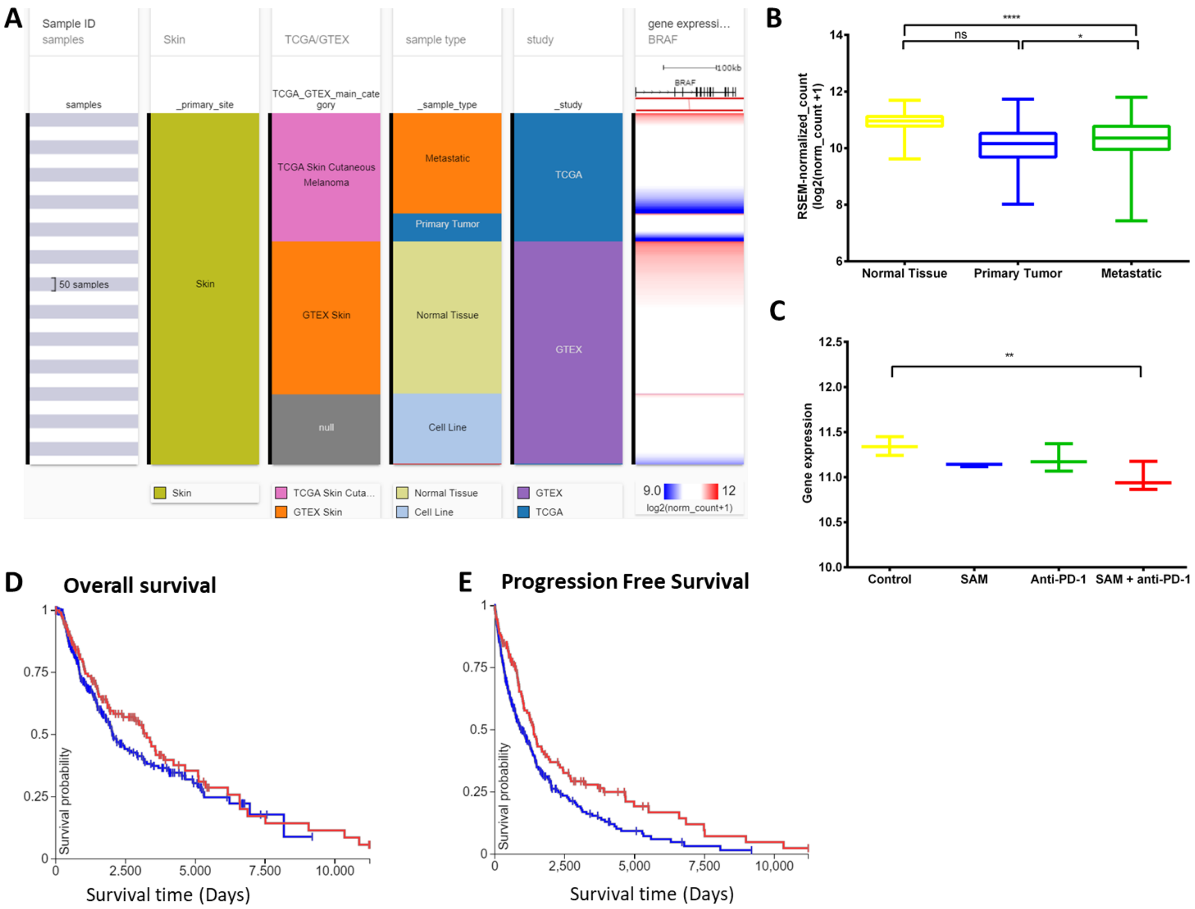


**Supplementary Figure 8: Clinical data analysis of *BRAF* gene identified from overlapping MGDB with RNA-seq data.** (A) Expression of *BRAF* gene in human healthy and skin cutaneous melanoma patients of GTEx and TCGA databases respectively. The columns show various phenotypic categories applied in order to stratify samples according to Sample Id, Skin (true), TCGA/GTEX, sample type (normal tissue, primary tumor, metastatic tissue or cell line) and study. The last column shows gene expression of *BRAF* of samples stratified according to the previous columns. Each row contains data from a single sample. (B) The expression data of *BRAF* in the normal tissue, primary tumor, metastatic tissue samples in (A) has been plotted in a Box-plot graph (n=1024 samples). Expression values are in RSEM (RNA-Seq by Expectation Maximization). (C) The expression data of *Braf* from RNA-sequencing of the primary B16 tumors after treatment with SAM, Anti-PD-1 and combination in this study (n =12; 3/group). Expression values are DeSEq2 normalized counts. (D-E) Overall survival and progression-free survival Kaplan Meier curves of *BRAF* from RNA-seq of GTEx and TCGA databases; X-axis: survival time (days); Y-axis: survival probability. (D) Low (blue) n= 327; High (red) n = 128. (E) Low (blue) n= 328; High (red) n = 128; P = **. Statistical significance was obtained by ANOVA in GraphPad prism and are represented by asterisks (**P < 0.05; **P < 0.01, ***P < 0.001 and ****P < 0.0001*). All the data and Supplementary Figure s, except (C), were generated using The UCSC Xena platform.

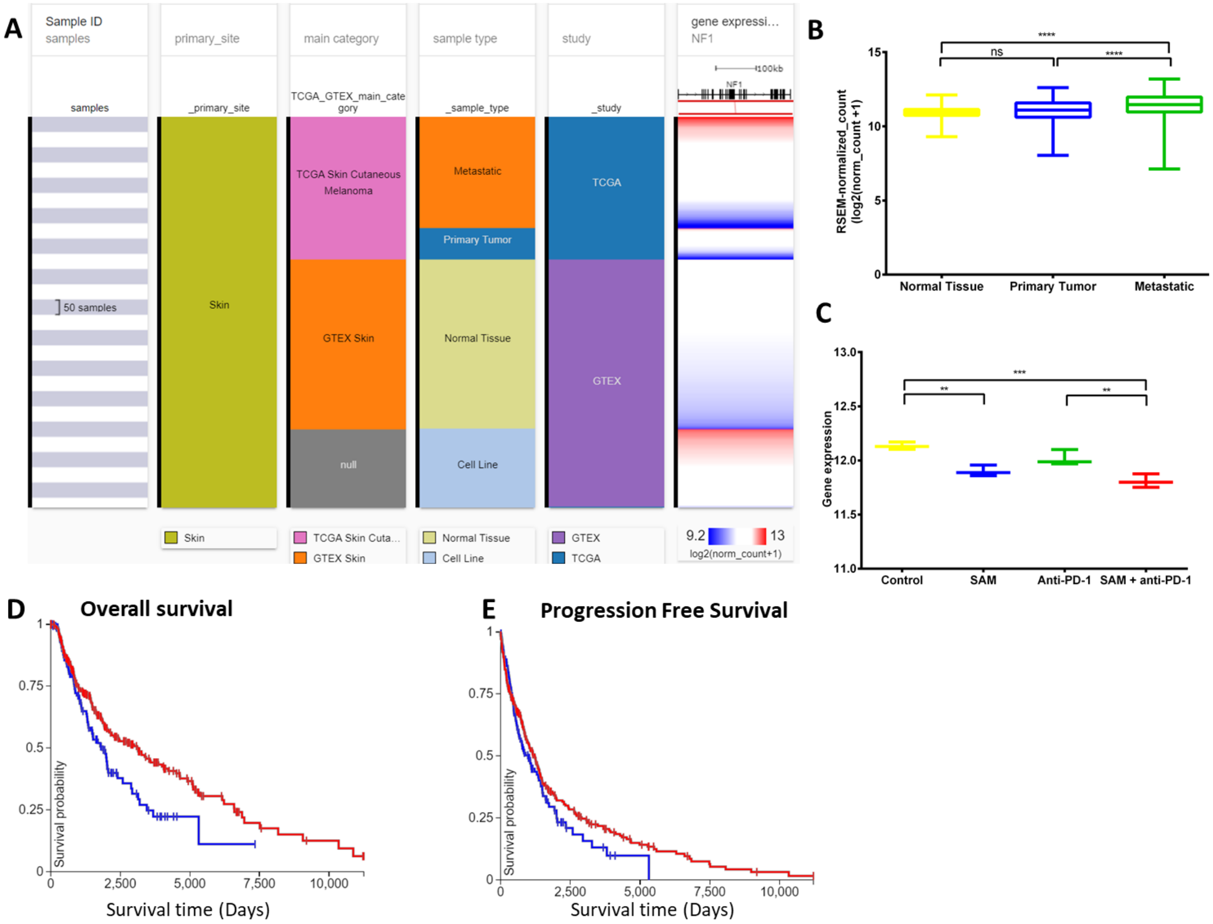


**Supplementary Figure 9: Clinical data analysis of *NF1* gene identified from overlapping MGDB with RNA-seq data.** (A) Expression of *NF1* gene in human healthy and skin cutaneous melanoma patients of GTEx and TCGA databases respectively. The columns show various phenotypic categories applied in order to stratify samples according to Sample Id, Skin (true), TCGA/GTEX, sample type (normal tissue, primary tumor, metastatic tissue or cell line) and study. The last column shows gene expression of *NF1* of samples stratified according to the previous columns. Each row contains data from a single sample. (B) The expression data of *NF1* in the normal tissue, primary tumor, metastatic tissue samples in (A) has been plotted in a Box-plot graph (n=1024 samples). Expression values are in RSEM (RNA-Seq by Expectation Maximization). (C) The expression data of *Nf1* from RNA-sequencing of the primary B16 tumors after treatment with SAM, Anti-PD-1 and combination in this study (n =12; 3/group). Expression values are DeSEq2 normalized counts. (D-E) Overall survival and progression-free survival Kaplan Meier curves of *NF1* from RNA-seq of GTEx and TCGA databases; X-axis: survival time (days); Y-axis: survival probability. (D) Low (blue) n= 164; High (red) n = 291; P = ****. (E) Low (blue) n= 165; High (red) n = 291. Statistical significance was obtained by ANOVA in GraphPad prism and are represented by asterisks (**P < 0.05; **P < 0.01, ***P < 0.001 and ****P < 0.0001*). All the data and Supplementary Figure s, except (C), were generated using The UCSC Xena platform.

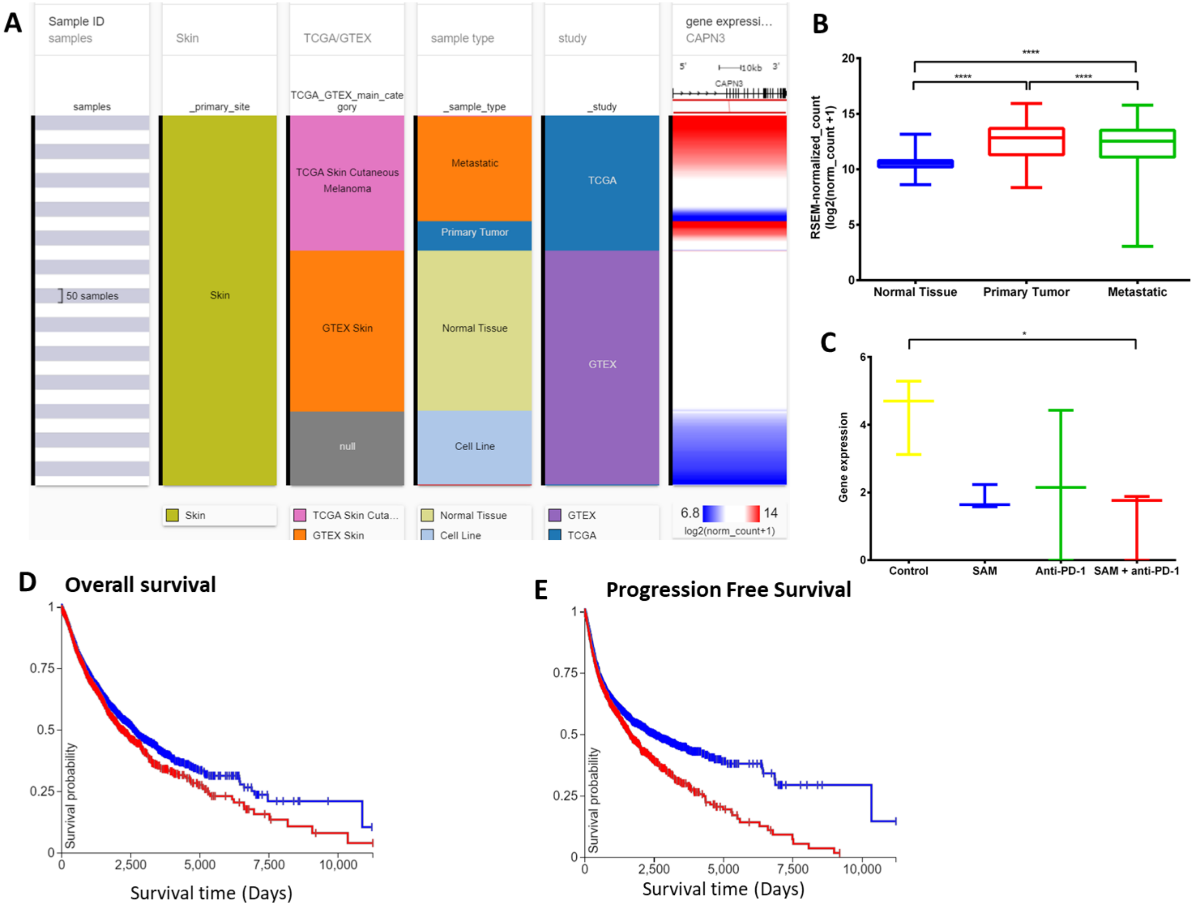


**Supplementary Figure 10: Analysis of *CAPN3* gene expression in clinical public data. (A)** Expression of *CAPN3* gene in human healthy and skin cutaneous melanoma patients of GTEx and TCGA databases respectively. The columns show various phenotypic categories applied in order to stratify samples according to Sample Id, Skin (true), TCGA/GTEX, sample type (normal tissue, primary tumor, metastatic tissue or cell line) and study. The last column shows gene expression of *CAPN3* of samples stratified according to the previous columns. Each row contains data from a single sample. **(B)** The expression data of *CAPN3* in the normal tissue, primary tumor, metastatic tissue samples in (A) has been plotted in a Box-plot graph (n=1024 samples). Expression values are in RSEM (RNA-Seq by Expectation Maximization). **(C)** The expression data of *Capn3* from RNA-sequencing of the primary B16 tumors after treatment with SAM, Anti-PD-1 and combination in this study (n =12; 3/group). Expression values are DeSEq2 normalized counts. (D-E) Overall survival and progression-free survival Kaplan Meier curves of CAPN3 from RNA-seq of GTEx and TCGA databases; X-axis: survival time (days); Y-axis: survival probability. **(D)** P=****; Log-rank test statistics = 8.356; Low (blue) n= 8098; High (red) n = 2336. (E) P = ****; Log-rank test statistics = 29.36; Low (blue) n= 8054; High (red) n = 2218. Statistical significance was obtained by ANOVA in GraphPad prism and are represented by asterisks (**P < 0.05; **P < 0.01, ***P < 0.001 and ****P < 0.0001*). All the data and Supplementary Figure s, except (C), were generated using The UCSC Xena platform.

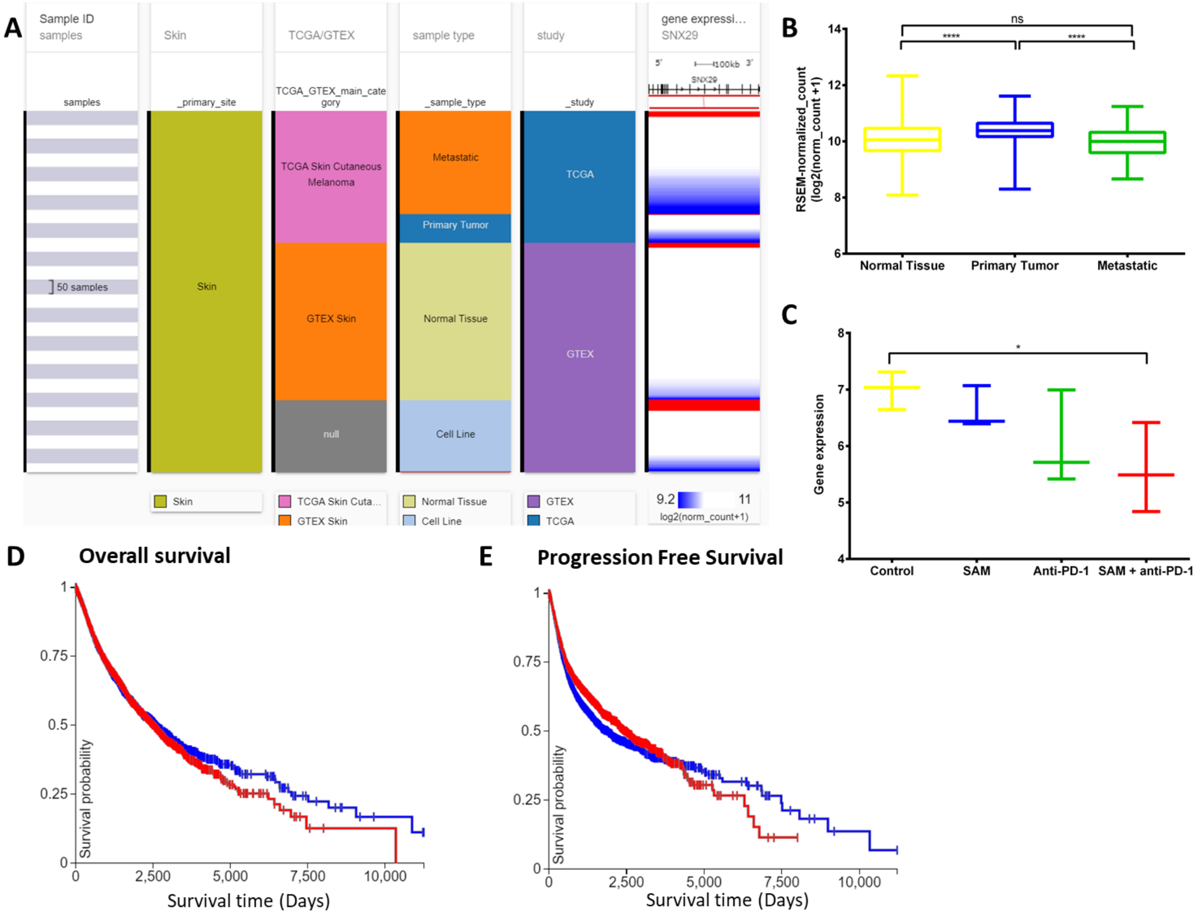


**Supplementary Figure 11: Clinical data analysis of *SNX29* gene identified from overlapping TCGA with RNA-seq data.** (A) Expression of *SNX29* gene in human healthy and skin cutaneous melanoma patients of GTEx and TCGA databases respectively. The columns show various phenotypic categories applied in order to stratify samples according to Sample Id, Skin (true), TCGA/GTEX, sample type (normal tissue, primary tumor, metastatic tissue or cell line) and study. The last column shows gene expression of *SNX29* of samples stratified according to the previous columns. Each row contains data from a single sample. (B) The expression data of *SNX29* in the normal tissue, primary tumor, metastatic tissue samples in (A) has been plotted in a Box-plot graph (n=1024 samples). Expression values are in RSEM (RNA-Seq by Expectation Maximization). (C) The expression data of *Snx29* from RNA-sequencing of the primary B16 tumors after treatment with SAM, Anti-PD-1 and combination in this study (n =12; 3/group). Expression values are DeSEq2 normalized counts. (D-E) Overall survival and progression-free survival Kaplan Meier curves of *SNX29* from RNA-seq of GTEx and TCGA databases; X-axis: survival time (days); Y-axis: survival probability. (D) Low (blue) n= 5156; High (red) n = 5278. (E) Low (blue) n= 5155; High (red) n = 5117; P = ****. Statistical significance was obtained by ANOVA in GraphPad prism and are represented by asterisks (**P < 0.05; **P < 0.01, ***P < 0.001 and ****P < 0.0001*). All the data and Supplementary Figure s, except (C), were generated using The UCSC Xena platform.

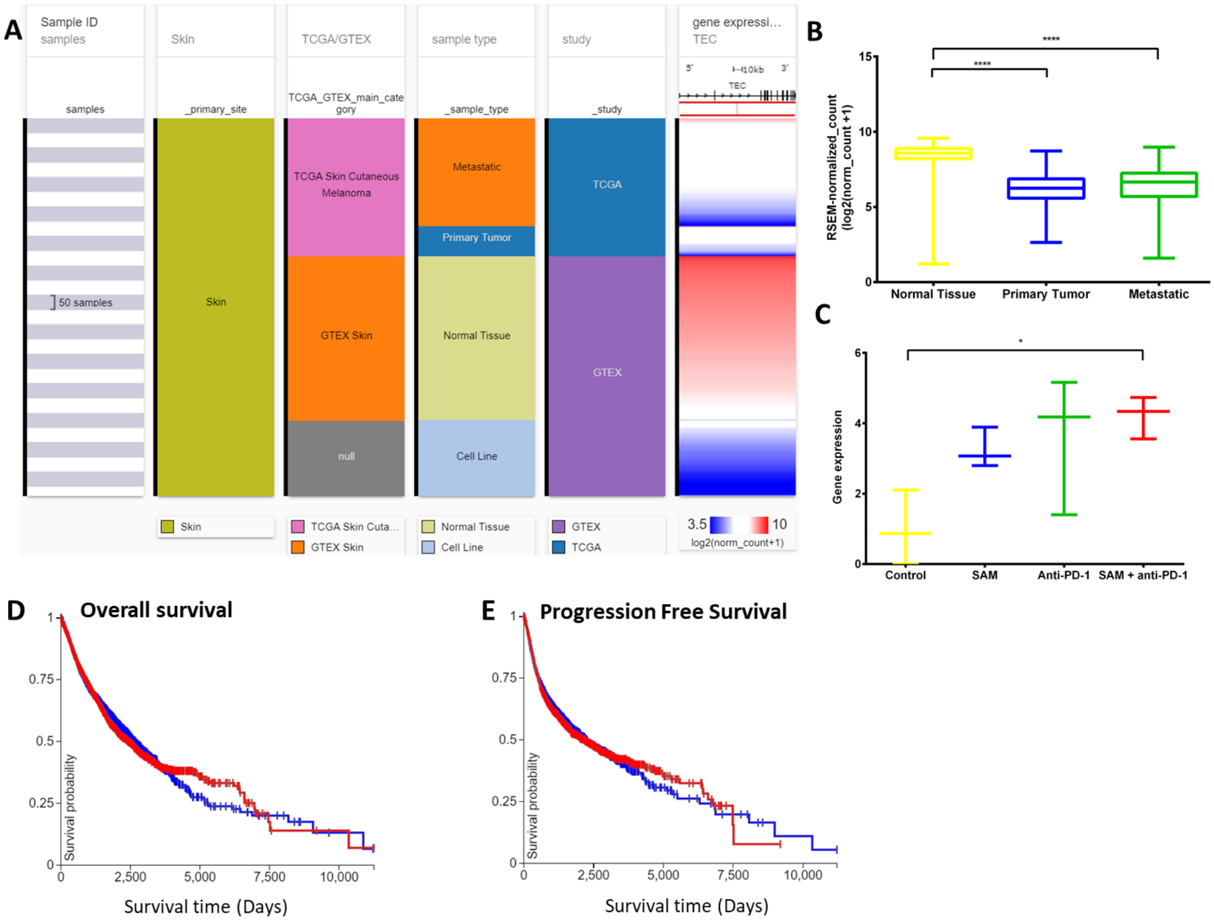


**Supplementary Figure 12: Clinical data analysis of *TEC* gene identified from overlapping TCGA with RNA-seq data.** (A) Expression of *TEC* gene in human healthy and skin cutaneous melanoma patients of GTEx and TCGA databases respectively. The columns show various phenotypic categories applied in order to stratify samples according to Sample Id, Skin (true), TCGA/GTEX, sample type (normal tissue, primary tumor, metastatic tissue or cell line) and study. The last column shows gene expression of *TEC* of samples stratified according to the previous columns. Each row contains data from a single sample. (B) The expression data of *TEC* in the normal tissue, primary tumor, metastatic tissue samples in (A) has been plotted in a Box-plot graph (n=1024 samples). Expression values are in RSEM (RNA-Seq by Expectation Maximization). (C) The expression data of mouse *TEC* from RNA-sequencing of the primary B16 tumors after treatment with SAM, Anti-PD-1 and combination in this study (n =12; 3/group). Expression values are DeSEq2 normalized counts. (D-E) Overall survival and progression-free survival Kaplan Meier curves of *TEC* from RNA-seq of GTEx and TCGA databases; X-axis: survival time (days); Y-axis: survival probability. (D) Low (blue) n= 5180; High (red) n = 5254. (E) Low (blue) n= 5179; High (red) n = 5093. Statistical significance was obtained by ANOVA in GraphPad prism and are represented by asterisks (**P < 0.05; **P < 0.01, ***P < 0.001 and ****P < 0.0001*). All the data and Supplementary Figure s, except (C), were generated using The UCSC Xena platform.

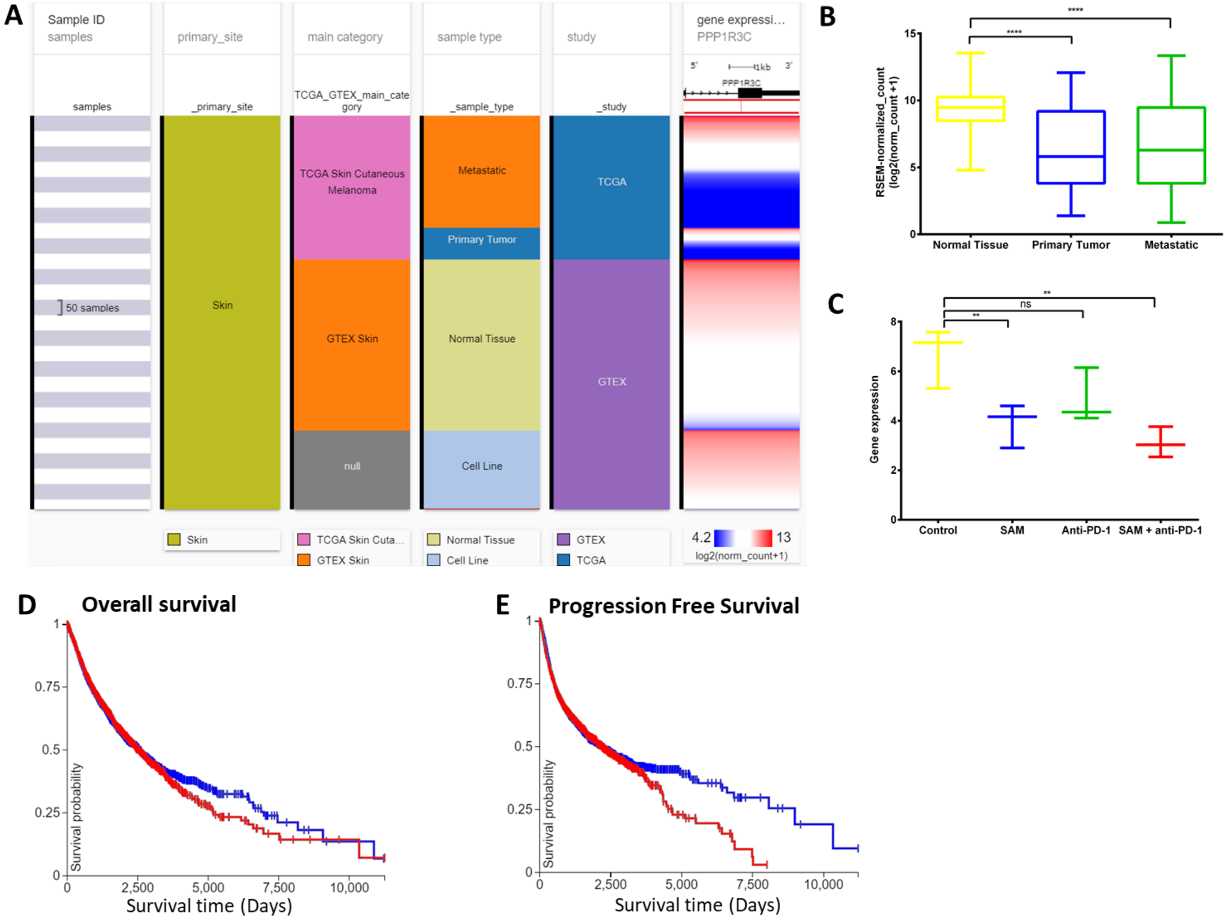


**Supplementary Figure 13: Clinical data analysis of *PPP1R3C* gene identified from overlapping MGDB with RNA-seq data.** (A) Expression of *PPP1R3C* gene in human healthy and skin cutaneous melanoma patients of GTEx and TCGA databases respectively. The columns show various phenotypic categories applied in order to stratify samples according to Sample Id, Skin (true), TCGA/GTEX, sample type (normal tissue, primary tumor, metastatic tissue or cell line) and study. The last column shows gene expression of *PPP1R3C* of samples stratified according to the previous columns. Each row contains data from a single sample. (B) The expression data of *PPP1R3C* in the normal tissue, primary tumor, metastatic tissue samples in (A) has been plotted in a Box-plot graph (n=1024 samples). Expression values are in RSEM (RNA-Seq by Expectation Maximization). (C) The expression data of *Ppp1r3c* from RNA-sequencing of the primary B16 tumors after treatment with SAM, Anti-PD-1 and combination in this study (n =12; 3/group). Expression values are DeSEq2 normalized counts. (D-E) Overall survival and progression-free survival Kaplan Meier curves of *PPP1R3C* from RNA-seq of GTEx and TCGA databases; X-axis: survival time (days); Y-axis: survival probability. (D) Low (blue) n= 6269; High (red) n = 4165. (E) Low (blue) n= 6108; High (red) n = 4164. Statistical significance was obtained by ANOVA in GraphPad prism and are represented by asterisks (**P < 0.05; **P < 0.01, ***P < 0.001 and ****P < 0.0001*). All the data and Supplementary Figure s, except (C), were generated using The UCSC Xena platform.

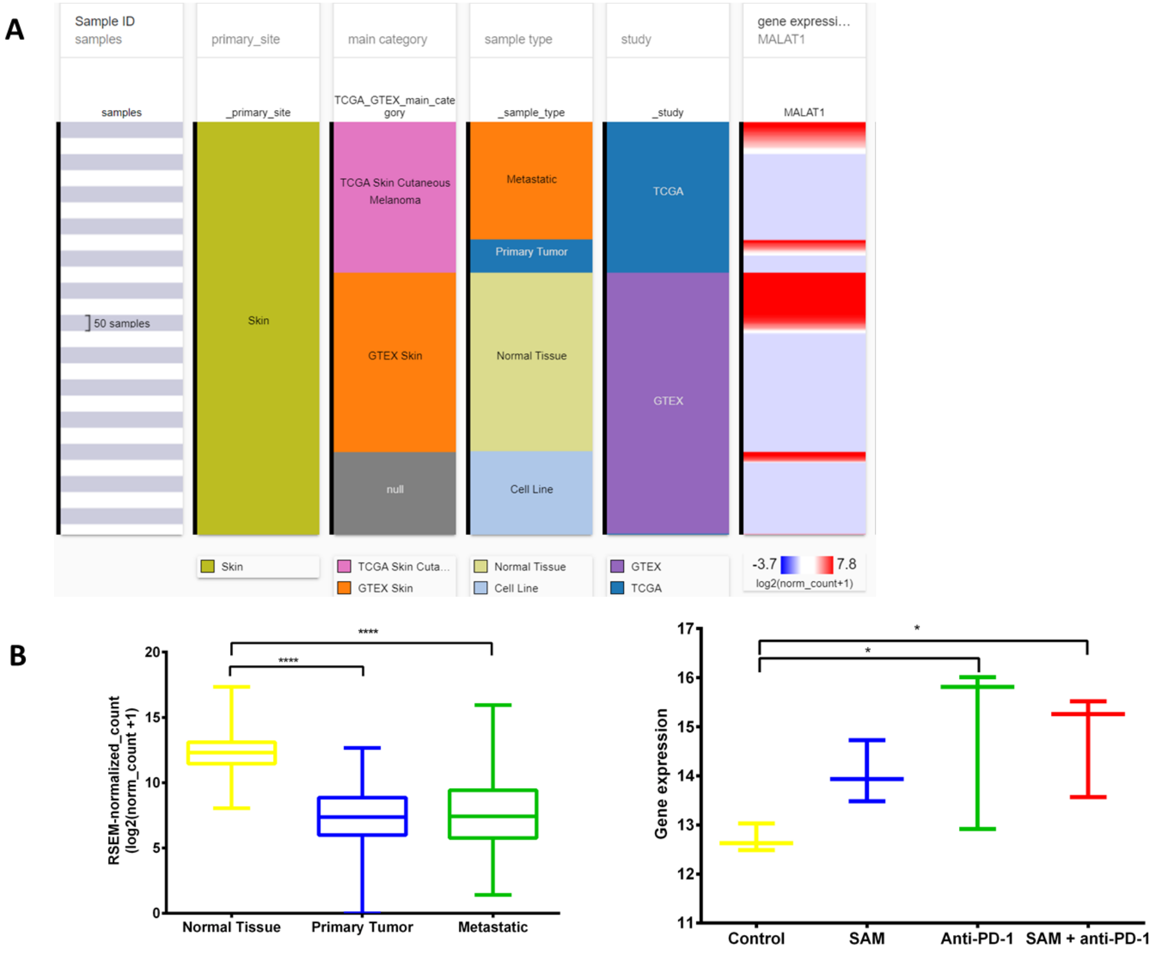


**Supplementary Figure 14: Clinical data analysis of a non-coding *MALAT1* gene identified from overlapping MGDB with RNA-seq data.** (A) Expression of *MALAT1* gene in human healthy and skin cutaneous melanoma patients of GTEx and TCGA databases respectively. The columns show various phenotypic categories applied in order to stratify samples according to Sample Id, Skin (true), TCGA/GTEX, sample type (normal tissue, primary tumor, metastatic tissue or cell line) and study. The last column shows gene expression of *MALAT1*of samples stratified according to the previous columns. Each row contains data from a single sample. (B) The expression data of *MALAT1* in the normal tissue, primary tumor, metastatic tissue samples in (A) has been plotted in a Box-plot graph. Expression values are in RSEM (RNA-Seq by Expectation Maximization). (C) The expression data of *Malat1* from RNA-sequencing of the primary B16 tumors after treatment with SAM, Anti-PD-1 and combination in this study (n =12; 3/group). Expression values are DeSEq2 normalized counts. Statistical significance was obtained by ANOVA in GraphPad prism and are represented by asterisks (*$*P < 0.05$; $**P < 0.01$, $***P < 0.001$ and $****P < 0.0001$*). All the data and Supplementary Figure s, except (C), were generated using The UCSC Xena platform.

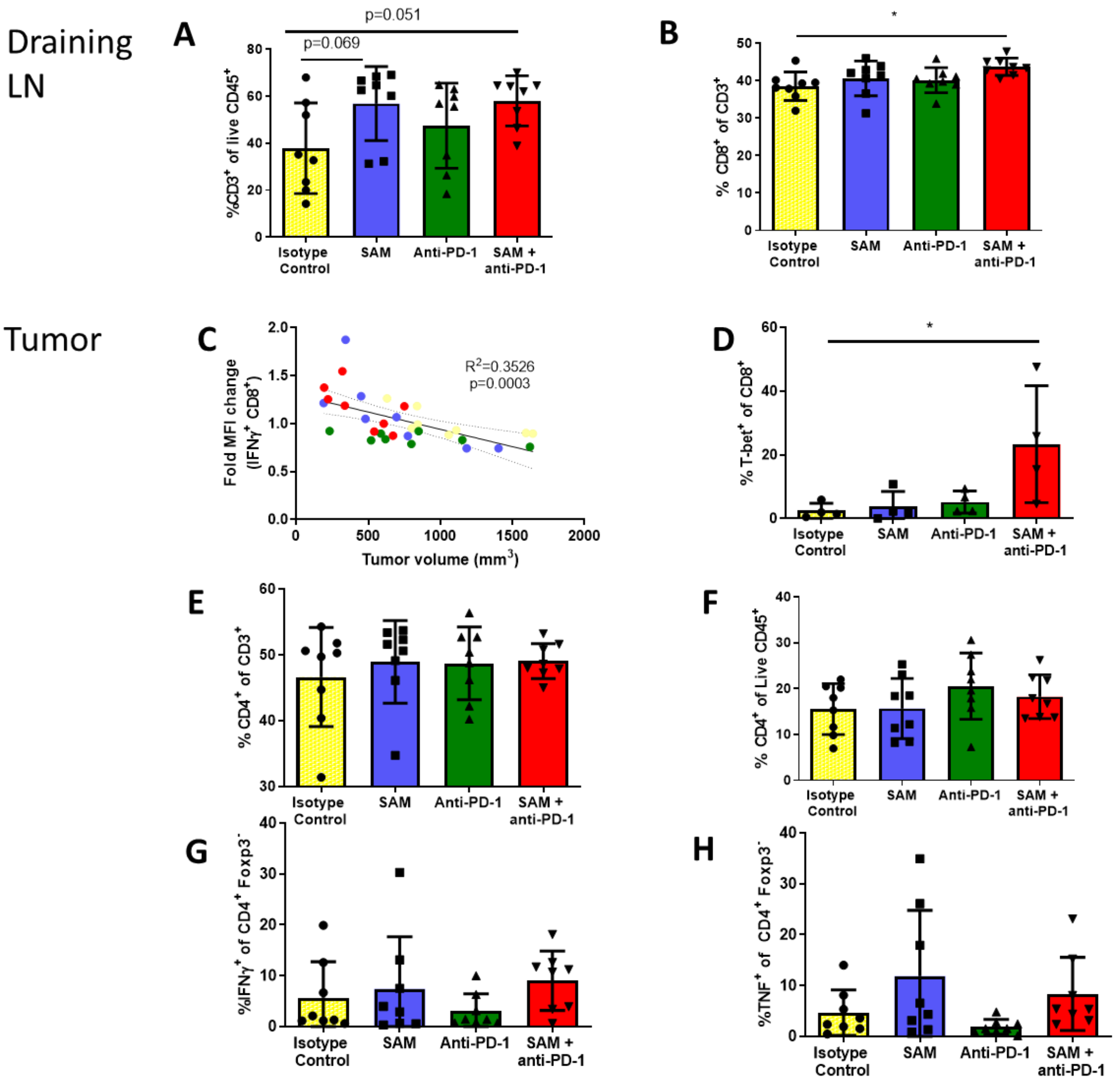


**Supplementary Figure 15: Effect of SAM, anti-PD-1 and SAM+anti-PD-1 on immune system in tumor micro-environment and draining lymph nodes (LN) as determined by tumor immuno-phenotyping using flow cytometry.** Treatment experiment had 4 groups; isotype IgG control, SAMe, anti-PD-1 and SAMe+anti-PD-1. (**A and B**) Percentage (%) of CD3+ cells in live CD45+ cells (checked from viability dye) and % of CD8+ of CD3+ T cells in the draining LN of mice treated with SAMe, anti-PD-1 and SAMe+anti-PD-1. (**C**) Correlation analysis of mean fluorescence intensity (MFI) of IFN-γ+ CD8+ T cells against tumor volume (mm3) of all the mouse in the 4 groups tested. (**D**) Percentage of T-bet+ CD8+ T cells in all the groups tested. (**E-F**) Percentage (%) of CD4+ T cells in CD3+ and in live CD45+ cells (checked from viability dye), respectively, in the primary tumors of mice in the 4 treatment groups. (**G-H**) Percentage of IFNγ+ and TNFα+ analysed in CD4+ Foxp3- T cells in all 4 groups. Statistical analysis was performed by ANOVA in GraphPad prism are represented by asterisks *(*P < 0.05*; ***P < 0.01*, and ****P < 0.001*).

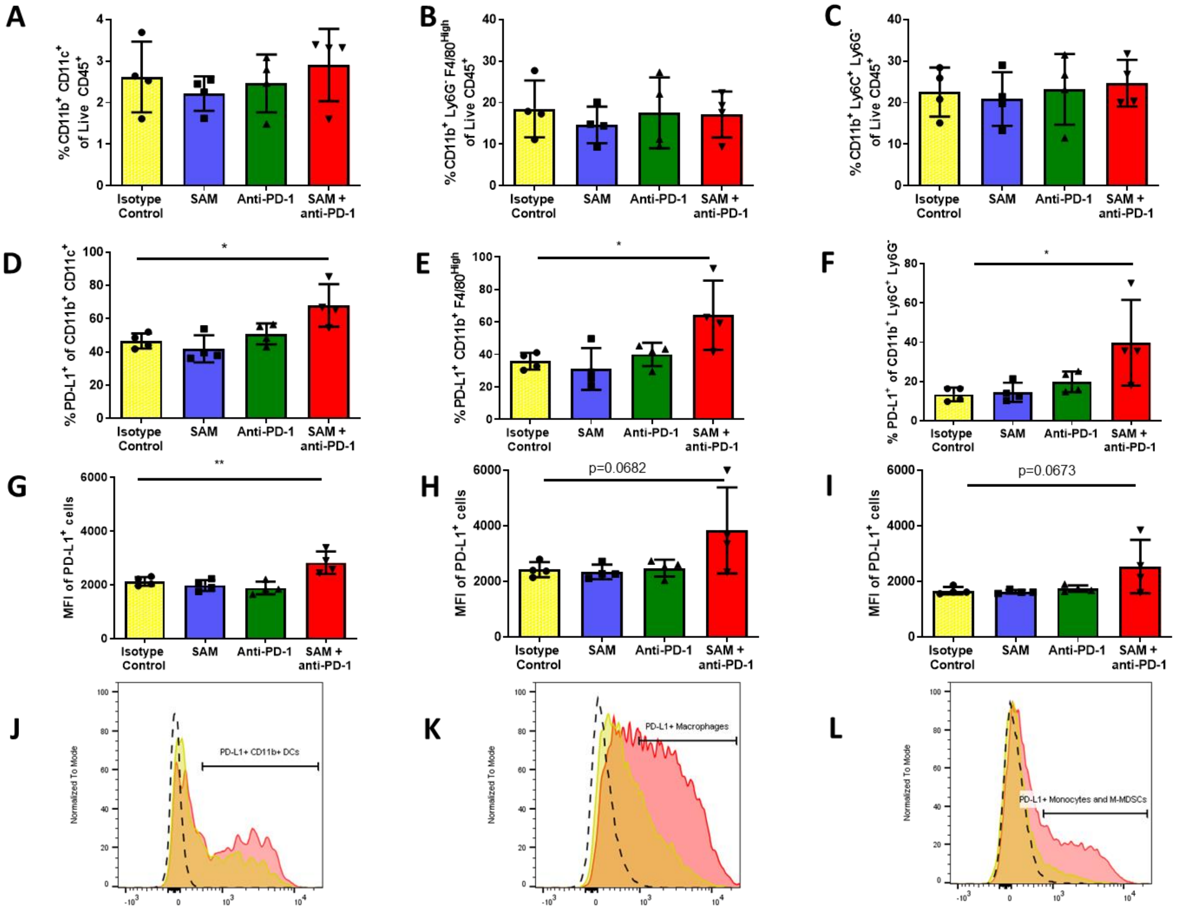


**Supplementary Figure 16: Effect of SAM, anti-PD-1 and SAM+anti-PD-1 on myeloid cells of the immune system in tumor micro-environment as determined by tumor immuno-phenotyping using flow cytometry.**

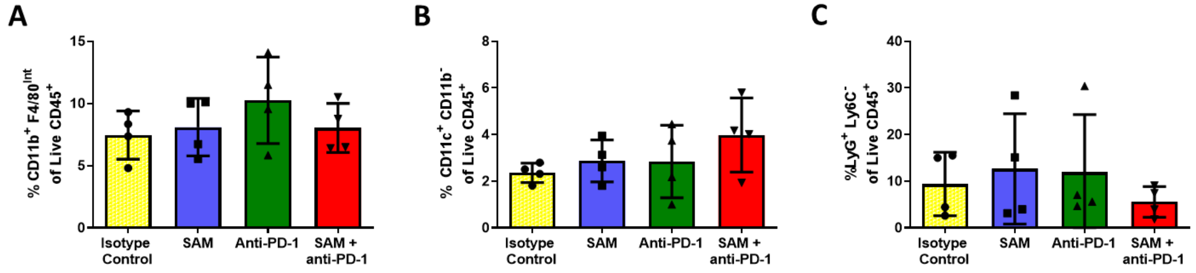


**Supplementary Figure 17: Effect of SAM, anti-PD-1 and SAM+anti-PD-1 on myeloid cells of the immune system in tumor micro-environment as determined by tumor immuno-phenotyping using flow cytometry.**

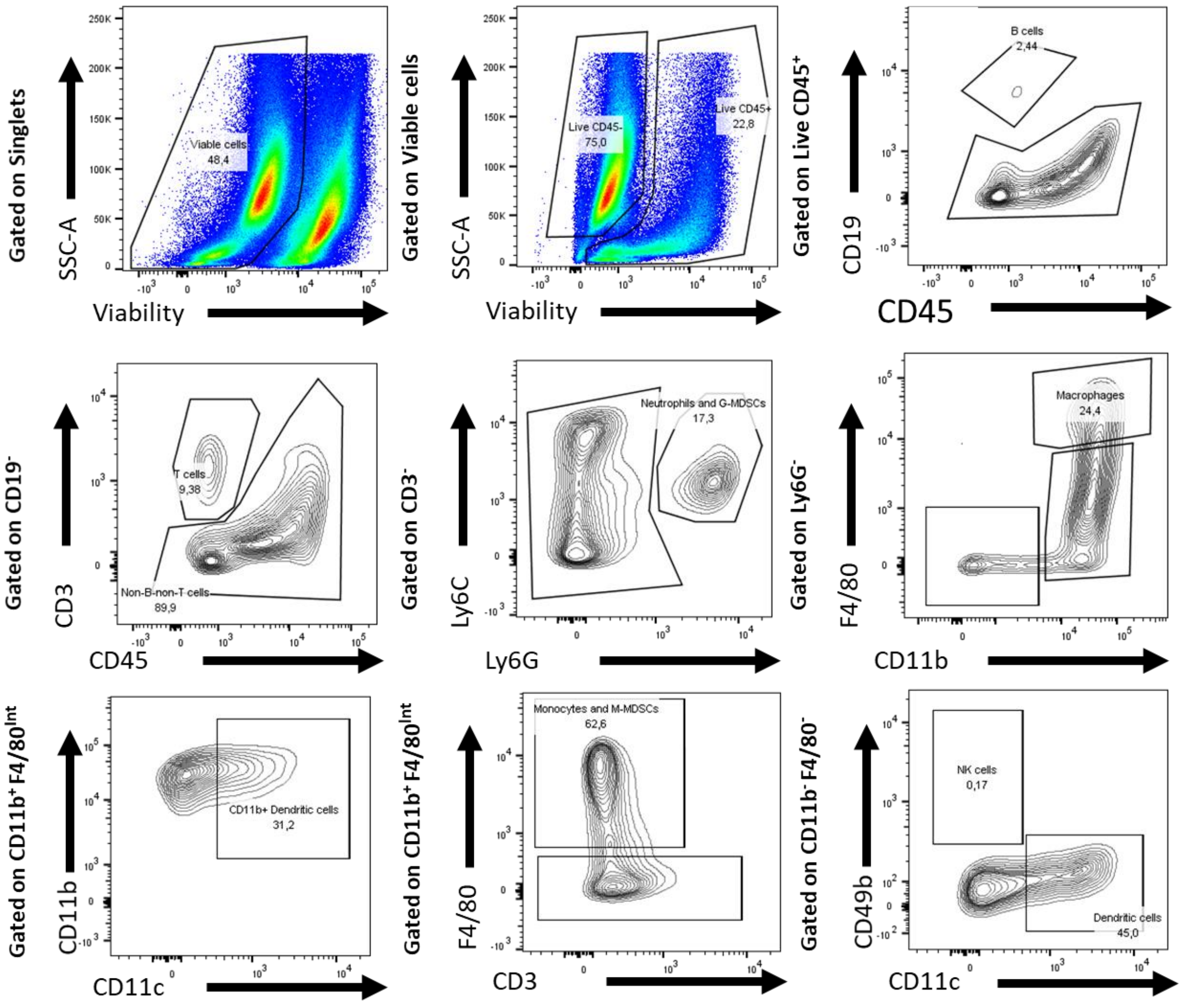


**Supplementary Figure 18: Representative gating strategy utilized for examining cell surface markers of myeloid cells in primary tumor of mice using flow cytometry.** Example used here is for tumor of isotype control group.

## 2 Supplementary Tables

**Supplementary Table 1:** List of primers used for this study.

| Gene Name | Forward | Reverse |
|---|---|---|
| ***Areg*** | GGTCTTAGGCTCAGGCCATTA | CGCTTATGGTGGAAACCTCTC |
| ***Fcgbp*** | AGTGGAAGGTCAAGGTGAACG | CAGGCCAAAGTCGGTTTCAAT |
| ***Myh2*** | AAGTGACTGTGAAAACAGAAGCA | GCAGCCATTTGTAAGGGTTGAC |

| | | |
|---|---|---|
| ***Xirp1*** | GCTCCGGCGTCTCTACAAAC | CCAGCGCATACACTGAACATC |
| ***Mybpc1*** | ATGGAATGGTTCACCGTCATTG | TAGTTGCATCCTCGCTAAGGC |
| ***Mybph*** | CAGCCACTAAGCCTGAACCTC | TCCAACACATAGCCTTGAAGC |
| ***Sypl2*** | CGCACCTCGGACAAGTCTC | CCCGAAGGCGAAAATAGCAAA |
| ***Dmbt1*** | TCAGCACAAGTCCTCCATCAT | TCCACAGGTGAGACTCATACC |
| ***Nrp2*** | GCTGGCTACATCACTTCCCC | CAATCCACTCACAGTTCTGGTG |

**Supplementary Table 2:** List of antibodies used for immunophenotyping along with their fluorophores.

| **T cell panels** | **Cytokine panel** | **Myeloid panel** |
|---|---|---|
| Fixable Viability Dye eFluor 780 (eBioscience) | V450 – Ki67 (clone B56, BD) | BUV395 – CD45.2 (clone104, eBioscience) |
| BUV737 – CD3 (clone 17A2, BD) | PE-Cy7 IFNγ (clone XMG1.2, BD) | FITC – CD3 (clone 17A2, BD) |
| Alexa Fluor 700 – CD4 (clone GK1.5, ThermoFisher) | BUV737 – IFNγ (clone XMG1.2, BD) | BUV737 – CD19 (clone 1D3, BD) |
| BV510 – CD8b (clone H35-17.2, BD) | PE-Cy7 – T-bet (clone 4B10, ThermoFisher) | Fixable Viability Dye eFluor 506 (eBioscience) |
| PE – PD-1 (clone J43, BD) | APC – IL-2 (BD) | PE-Cy7 – F4/80 (clone BM8, eBioscience) |
| PE-Cy7 – CD25 (clone PC61, BD) | PerCP-Cy5.5 – TNF (clone MP6-XT22, BD) | PerCP-Cy5.5 – CD11c (clone HL3, BD) |
| PerCP-Cy5.5 – CD25 (clone PC61.5, BD) | AntiCD16/CD32 (clone 2.4G2, BD) | Pacific Blue – CD11b (clone M1/70, eBioscience) |
| APC- ICOS (clone C396.4A, Thermofisher) | | PE- CD49b (clone DX5, BD) |
| PE-Cy7 – CTLA-4 (clone UC-4B9, Biolegend) | | Alexa Fluor 700 – Ly6G (clone 1A8, BioLegend) |
| APC – PD-L1 (clone MIH5, BD) | | APC-780 – Ly6C (clone AL21, BD) |
| FITC – Foxp3 (clone FJK16s, eBioscience) | | AntiCD16/CD32 (clone 2.4G2, BD) |
| BUV395 – CD45 (clone 104, BD) | | |
